# Investigation of the dynamical structures of double-chain deoxyribonucleic acid model in biological sciences

**DOI:** 10.1038/s41598-024-55786-z

**Published:** 2024-03-17

**Authors:** Muhammad Zain Yousaf, Muhammad Abbas, Tahir Nazir, Farah Aini Abdullah, Asnake Birhanu, Homan Emadifar

**Affiliations:** 1 Department of Mathematics, University of Sargodha, 40100 Sargodha, Pakistan; 2 School of Mathematical Sciences, Universiti Sains Malaysia, 11800 Penang, Malaysia; 3 Department of Mathematics, College of Science, Hawassa University, Hawassa, Ethiopia; 4 Department of Mathematics, Saveetha School of Engineering, Saveetha Institute of Medical and Technical Sciences, Saveetha University, Chennai, Tamil Nadu, 602 105 India; 5 MEU Research Unit, Middle East University, Amman, Jordan

**Keywords:** Modified extended Fan sub equation approach, Double-chain deoxyribonucleic acid model, Exact traveling wave solutions, 3D graph, 2D line graph, Contour plot, Biological models, Mathematics and computing, Applied mathematics

## Abstract

The present research investigates the double-chain deoxyribonucleic acid model, which is important for the transfer and retention of genetic material in biological domains. This model is composed of two lengthy uniformly elastic filaments, that stand in for a pair of polynucleotide chains of the deoxyribonucleic acid molecule joined by hydrogen bonds among the bottom combination, demonstrating the hydrogen bonds formed within the chain’s base pairs. The modified extended Fan sub equation method effectively used to explain the exact travelling wave solutions for the double-chain deoxyribonucleic acid model. Compared to the earlier, now in use methods, the previously described modified extended Fan sub equation method provide more innovative, comprehensive solutions and are relatively straightforward to implement. This method transforms a non-linear partial differential equation into an ODE by using a travelling wave transformation. Additionally, the study yields both single and mixed non-degenerate Jacobi elliptic function type solutions. The complexiton, kink wave, dark or anti-bell, V, anti-Z and singular wave shapes soliton solutions are a few of the creative solutions that have been constructed utilizing modified extended Fan sub equation method that can offer details on the transversal and longitudinal moves inside the DNA helix by freely chosen parameters. Solitons propagate at a consistent rate and retain their original shape. They are widely used in nonlinear models and can be found everywhere in nature. To help in understanding the physical significance of the double-chain deoxyribonucleic acid model, several solutions are shown with graphics in the form of contour, 2D and 3D graphs using computer software Mathematica 13.2. All of the requisite constraint factors that are required for the completed solutions to exist appear to be met. Therefore, our method of strengthening symbolic computations offers a powerful and effective mathematical tool for resolving various moderate nonlinear wave problems. The findings demonstrate the system’s potentially very rich precise wave forms with biological significance. The fundamentals of double-chain deoxyribonucleic acid model diffusion and processing are demonstrated by this work, which marks a substantial development in our knowledge of double-chain deoxyribonucleic acid model movements.

## Introduction

Each cell’s primary means of storing data is deoxyribonucleic acid. It resembles a very significant straight molecule that holds the inherited instructions that distinguish one species from the others in the sort of structure of a nucleotide chain. Because its significance with the origins of existence, the structure and function of DNA molecules have become one of the foremost fascinating issues in the discipline of contemporary biophysics. Because it consists of two complimentary chains or strands, DNA is dual-stranded. Since both of these chains are joined by bonds of hydrogen and operate counter to one another, they are referred to as oppositely strands. In DNA, the term “nucleic acid” speaks to the phosphate particles, whereas “deoxyribo” defines the sugar.

The field of genetics evolved in 1953 when Watson and Crick identified the framework of the double-chain deoxyribonucleic acid (D-CDNA) model. More significantly, the double helix concept contributes to the understanding of the recurrence DNA process in addition to indicating the known layout of DNA. Nonlinear (NL) science is being applied to analyzing D-CDNA frameworks in order to understand further about their characteristics. This is why the characteristics of D-CDNA can be examined using NL models that combine biological tools and scientific methodologies. When attempting to investigate the configuration of D-CDNA from a NL science perspective, the appropriate NL mathematical models must be identified. The intricate layout of the D-CDNA mechanism and the existence of distinct motions make it challenging to imitate at first using a particular mathematical model.

Since strands of DNA are the building blocks of life, their structure and function represent one of the most interesting biophysical mysteries of our day. Over the years, researchers have examined the makeup of DNA. Predicting the emergence of significant NL structures has proved effective in the study of DNA dynamics. It has been demonstrated that nonlinearity causes confined waves. The capacity of these confined waves to transfer energy with no dissipating makes them intriguing. Numerous scientists have attempted to create mathematical representations of the mathematical biology. Indeed, this is the one of the main reasons to take this model into consideration. Numerous scholars have created and employed a wide range of approaches in the literature for resolving these mathematical structures. As an illustration, F-expansion approach^1^, Hirota bilinear method^2,3^, Kudryashov technique^4^, translation method^5^, inverse scattering scheme^6^, homogeneous balance approach^7^, $\frac{G}{G'}$-expansion approach^8^, tanh-function approach^9^, fractional dual-function scheme^10^, exp-function approach^11^, sine cosine approach^12^, $\Phi ^{6}$-model expansion approach^13^, improved sardar sub-equation method^14^, generalized exponential rational function method^15^, direct algebraic technique^16^.

Numerous mathematicians and scientists have examined the dynamics of the D-CDNA model in^17–22^. $\phi ^{6}$-model expansion technique utilized in^23^ to analyze mathematical dynamics in the D-CDNA model. A helicoidal D-CDNA model’s semi-discrete breather is examined in^24^. A DNA structure is supplied in^25^ using both the wormlike-chain and bead models. The D-CDNA dynamical structure within the context of a pandemic is analyzed in^26^. The DNA model’s precise closed and solitonic structures can be reviewed in^27^. In order to calculate the circuitry of a D-CDNA, a tight-binding framework has been developed and provided in^28^. For the development of D-CDNA loops, a self-avoiding wormlike chain model is provided in^29^. In^30^, an unpredictable genome evolution in a D-CDNA system has been investigated using lie symmetry. The exact travelling wave (TW) solutions for NLDEs in a D-CDNA Model is demonstrated in^31^. In^32^, the behavior of soliton solutions in the D-CDNA model can be observed via two analytical techniques. The D-CDNA model has many accurate solutions that are closed-form utilising GERF provided in^33^. The exact analytical solution for PDE that can be achieved through the bilinear residual network and bilinear neural network methods^34–37^ is considerably better compared to the traditional neural network numerical method.

The research presented here used modified extended Fan sub equation method (MEFSEM) to construct the D-CDNA model’s exact TW solutions, which are crucial for biologically linked research. After reading through the literature, it is discovered that MEFSEM has not yet been used in the construction of exact TW solutions of the D-CDNA model. Consequently, with this navigate, we’re going to effectively employ the suggested technique and generate exact TW solutions for the D-CDNA model in this work. The come upon solutions are innovative and have prospective uses in NL sciences. Solitons have been utilized to investigate several significant real-world problems across domains including fluid dynamics, plasma, nonlinear optics, astrophysics, and molecular biology.

As an illustration, the conceptual framework of solitons has been specifically used in fiber optics to the long-range transfer of digital information. In addition to their implementation in communication, solitons are employed in optical converters. Despite its significant potential applications in technological innovation, optical solitons continue to be among the foremost study areas in soliton theory today. Numerous types of solitary waves have been employed in biology to explain low frequency collective motion in proteins and DNA, the nervous system, and the transmission of signals and energy in biomembranes. Additionally, as plasmas are made up of considerably highly charged particles, soliton research is important in this field as well. For instance, NL oscillator chains that permit a variety of solitary wave solutions have been utilized to describe dusty plasmas, which are composed of micro charged dust particles.

Analytical techniques and numerical methods are the primary areas of investigation for soliton solutions of various types for tackling NLPDEs. The MEFSEM, an inexpensive and effective analytical technique, is employed in this work. The foundation of this technique is a widely recognized approximation. By using MEFSEM, one can acquire well-formed solutions that provide immediate insight. Heavy computing, which naturally requires a lot of time and resources, is not necessary with this technique. But there are also drawbacks to adopting MEFSEM in biotechnology, such as time and expense, particularly when working with complicated products, massive manufacturing, and rapidly evolving markets. The first-order derivatives problems arise in many technical domains, including fluid dynamics, biological sciences, physics, and mechanics are also extremely challenging to solve employing MEFSEM.

The subsequent format demonstrates how this study is designed: A description of the suggested MEFSEM is provided in section “Algorithm of the MEFSEM”. Section “Mathematical formulation and implementation of the MEFSEM” explains the construction of the DNA model mathematically and the implementation of MEFSEM to it. Section “Graphical Findings and discussion” analyzes the visual behaviors of the exact TW solutions of the D-CDNA model using MEFSEM. A few endure observations are presented in section “Conclusion”.

## Algorithm of the MEFSEM

The procedure for the MEFSEM utilizing the generalized elliptic equation is provided in the this section. Below is an outline of the key steps of our methodology.

Examine the subsequent non-linear partial differential equation (NLPDE), which has two independent spatial and temporal variables: *s* and *t*.

1 $$\begin{aligned} A(\Theta ,{\Theta _s}, {\Theta _t}, {\Theta _{ss}},{\Theta _{tt}},{\Theta _{st}},\ldots ) = 0, \end{aligned}$$

where in above Eq. (1) $\Theta (s, t)$ is unknown function, A is the polynomial with $\Theta (s, t)$ and its partial derivatives indicated by the subscripts, which comprise NL factors and derivatives of the greatest order. In the following steps the contexts of the MEFSEM are described.

*Step 1* The creation of a single variable $\varrho$ from the separate spatial and temporal variables such that:

2 $$\begin{aligned} \Theta (s, t) = \Upsilon (\varrho ),\ \ \ \ \ \varrho = \xi s + \zeta t. \end{aligned}$$

To determine the TW solutions, interpret Eq. (1) to the succeeding ODE by employing the wave transformation provided in Eq. (2)

3 $$\begin{aligned} B(\Upsilon , \Upsilon ',\Upsilon '',\Upsilon ''',\ldots ) = 0, \end{aligned}$$

where B is a polynomial in $\Upsilon (\varrho )$ combine with derivatives of $\Upsilon (\varrho )$ and prime signifies the derivative in terms of $\varrho$ which means $\Upsilon '(\varrho )=\frac{d\Upsilon }{d\varrho }$, $\Upsilon ''(\varrho )=\frac{d^2 \Upsilon }{d\varrho ^2}$ and so on.

*Step 2* In the subsequent finite series format, the solution to ODE (3) is presumed

4 $$\begin{aligned} \Upsilon (\varrho ) = \sum \limits _{l = 0}^M {{\eta _{l}}{\chi ^l(\varrho )}}, \end{aligned}$$

where *M* is a numerical value which needs to be determined, and $\eta _l's$ are real constants with $\eta _M \ne 0$ to be identified. The function $\Upsilon (\varrho )$ is the following elliptic equation

5 $$\begin{aligned} \chi ^{'2}(\varrho )=c_{0}+c_{1}\chi (\varrho )+c_{2}\chi ^{2}(\varrho )+c_{3}\chi ^{3}(\varrho )+c_{4}\chi ^{4}(\varrho ), \end{aligned}$$

where $c_{0}, c_{1}, c_{2}, c_{3}$ and $c_{4}$ are constants that need to be established. Under certain situations, there can be three parameters $\sigma , \mu , \rho$ if $c_{0}, c_{1}, c_{2}, c_{3}$ and $c_{4} \ne 0$ such that

6 $$\begin{aligned} \chi ^{'2}(\varrho )=c_{0}+c_{1}\chi (\varrho )+c_{2}\chi ^{2}(\varrho )+c_{3}\chi ^{3}(\varrho )+c_{4}\chi ^{4}(\varrho )=(\sigma +\mu \chi (\varrho )+\rho \chi ^{2}(\varrho ))^{2}. \end{aligned}$$

Equation (6) is solely satisfied in the scenario that subsequent relations hold.

7 $$\begin{aligned} c_{0}=\sigma ^{3}, \ \ \ c_{1}=2\sigma \mu , \ \ \ c_{2}=2\rho \sigma +\mu ^{2}, \ \ \ c_{3}=2\mu \rho , \ \ \ c_{4}=\rho ^{2}. \end{aligned}$$

In certain instances, if $c_{0}, c_{1}, c_{3}$ and $c_{4} \ne 0$ and $c_{2}=0$, three factors $\sigma , \mu , \rho$ could possibly be present such that

8 $$\begin{aligned} \chi ^{'2}(\varrho )=c_{0}+c_{1}\chi (\varrho )+c_{3}\chi ^{3}(\varrho )+c_{4}\chi ^{4}(\varrho )=(\sigma +\mu \chi (\varrho )+\rho \chi ^{2}(\varrho ))^{2}. \end{aligned}$$

Equation (8) is solely satisfied in the scenario that subsequent relations hold.

9 $$\begin{aligned} c_{0}=\sigma ^{3}, \ \ \ c_{1}=2\sigma \mu , \ \ \ c_{3}=2\mu \rho , \ \ \ c_{4}=\rho ^{2}. \end{aligned}$$

Among $\sigma , \mu$ and $\rho$ parameters, the following constraint is needed to be present

10 $$\begin{aligned} \mu ^{2}=-2\sigma \rho , \ \ \ \ \ \sigma \rho < 0. \end{aligned}$$

Therefore the generalized Riccati equation (GRE) is obtained by simplifying the general elliptic equation (GEE) using Eqs. (6) and (8). This auxiliary equation is produced from the GEE for $c_{0} = c_{1} = 0$

11 $$\begin{aligned} \chi ^{'2}(\varrho )=c_{2}\chi ^{2}(\varrho )+c_{3}\chi ^{3}(\varrho )+c_{4}\chi ^{4}(\varrho ). \end{aligned}$$

The GEE generates the elliptic equation when $c_{1} = c_{3} = 0$

12 $$\begin{aligned} \chi ^{'2}(\varrho )=c_{0}+c_{2}\chi ^{2}(\varrho )+c_{4}\chi ^{4}(\varrho ), \end{aligned}$$

where in above Eq. (12) there is the Riccati equation.

13 $$\begin{aligned} \chi ^{'2}(\varrho )=(D+\chi ^{2}(\varrho ))^{2}. \end{aligned}$$

The GEE takes on its subsequent form when $c_{2} = c_{4} = 0$

14 $$\begin{aligned} \chi ^{'2}(\varrho )=c_{0}+c_{1}\chi (\varrho )+c_{3}\chi ^{3}(\varrho ). \end{aligned}$$

*Step 3* Through considering the homogeneous balance method between the linear and NL factors of the highest order given in Eq. (3), the positive integer *M* observe in solution (4) can easily obtained.

*Step 4* A polynomial in $\chi (\varrho )$ can be initiated through swapping the solution (4) into Eq. (3) with the support of Eq. (5). Next, combine each term that have the same $\chi (\varrho )$ powers. Thus, through applying the same powers of $\chi (\varrho )$ equal to zero, a system of algebraic equations can be generated. Mathematica 13.2 is used to solve these equations consequently, it is feasible to discover the crucial constant values $\eta _l's$. The exact TW solutions of Eq. (1) can be retrieved by inserting all constants and Eqs. (4)–(5) into Eq. (3).

*Special cases*

- *Case 1*
  - *Type 1* $$\mu ^{2}-4\rho \sigma >0$$, $$\mu \rho \ne 0$$ and $$\rho \sigma \ne 0$$:
    15 $$\begin{aligned}{} & {} {\left\{ \begin{array}{ll} \chi _{1}^{I}(\varrho )=-\frac{\mu +\sqrt{\mu ^{2}-4\rho \sigma } \tanh { \left( \frac{\sqrt{\mu ^{2}-4\rho \sigma }\varrho }{2} \right) }}{2\rho }{,}\\ \chi _{2}^{I}(\varrho )=-\frac{\mu +\sqrt{\mu ^{2}-4\rho \sigma } \coth { \left( \frac{\sqrt{\mu ^{2}-4\rho \sigma }\varrho }{2} \right) }}{2\rho }{;} \end{array}\right. } \end{aligned}$$
    16 $$\begin{aligned}{} & {} {\left\{ \begin{array}{ll} \chi _{3}^{I}(\varrho )=-\frac{\mu +\sqrt{\mu ^{2}-4\rho \sigma } \left( \tanh \left( {\sqrt{\mu ^{2}-4\rho \sigma }\varrho } \right) \pm \iota \text {sech} \left( {\sqrt{\mu ^{2}-4\rho \sigma }\varrho } \right) \right) }{2\rho }{,}\\ \chi _{4}^{I}(\varrho )=-\frac{\mu +\sqrt{\mu ^{2}-4\rho \sigma } \left( \coth \left( {\sqrt{\mu ^{2}-4\rho \sigma }\varrho } \right) \pm \text {csch} \left( {\sqrt{\mu ^{2}-4\rho \sigma }\varrho } \right) \right) }{2\rho }{,}\\ \chi _{5}^{I}(\varrho )=-\frac{2\mu +\sqrt{\mu ^{2}-4\rho \sigma } \left( \tanh { \left( \frac{\sqrt{\mu ^{2}-4\rho \sigma }}{4}\varrho \right) }+\coth { \left( \frac{\sqrt{\mu ^{2}-4\rho \sigma }}{4}\varrho \right) } \right) }{4\rho }{;} \end{array}\right. } \end{aligned}$$
    17 $$\begin{aligned}{} & {} {\left\{ \begin{array}{ll} \chi _{6}^{I}(\varrho )=\frac{1}{2\rho }(-\mu +\frac{\sqrt{(E^{2}+F^{2})(\mu ^{2}-4\rho \sigma )}-E(\sqrt{\mu ^{2}-4\rho \sigma })\cosh (\sqrt{\mu ^{2}-4\rho \sigma }\varrho )}{E\sinh (\sqrt{\mu ^{2}-4\rho \sigma }\varrho )+F}){,}\\ \chi _{7}^{I}(\varrho )=\frac{1}{2\rho }(-\mu -\frac{\sqrt{(F^{2}-E^{2})(\mu ^{2}-4\rho \sigma )}+E(\sqrt{\mu ^{2}-4\rho \sigma })\sinh (\sqrt{\mu ^{2}-4\rho \sigma }\varrho )}{E\cosh (\sqrt{\mu ^{2}-4\rho \sigma }\varrho )+F}){;} \end{array}\right. } \end{aligned}$$
    where E and F are non-zero constants that fulfill $F^{2}-E^{2}>0$.
    18 $$\begin{aligned}{} & {} {\left\{ \begin{array}{ll} \chi _{8}^{I}(\varrho )= \frac{2\sigma \cosh { \left( \frac{\sqrt{\mu ^{2}-4\rho \sigma }\varrho }{2} \right) }}{\sqrt{\mu ^{2}-4\rho \sigma }\sinh {\left( \frac{\sqrt{\mu ^{2}-4\rho \sigma }\varrho }{2} \right) }-\mu \cosh { \left( \frac{\sqrt{\mu ^{2}-4\rho \sigma }\varrho }{2} \right) }}{,}\\ \chi _{9}^{I}(\varrho )= \frac{-2\sigma \sinh { \left( \frac{\sqrt{\mu ^{2}-4\rho \sigma }\varrho }{2} \right) }}{\mu \sinh {\left( \frac{\sqrt{\mu ^{2}-4\rho \sigma }\varrho }{2} \right) }-\sqrt{\mu ^{2}-4\rho \sigma }\cosh {\left( \frac{\sqrt{\mu ^{2}-4\rho \sigma }\varrho }{2} \right) }}{;} \end{array}\right. } \end{aligned}$$
    19 $$\begin{aligned}{} & {} {\left\{ \begin{array}{ll} \chi _{10}^{I}(\varrho )= \frac{2\sigma \cosh { \left( \sqrt{\mu ^{2}-4\rho \sigma }\varrho \right) }}{\sqrt{\mu ^{2}-4\rho \sigma }\sinh { \left( \sqrt{\mu ^{2}-4\rho \sigma }\varrho \right) }- \left( \mu \cosh { \left( \sqrt{\mu ^{2}-4\rho \sigma }\varrho \right) }\pm \iota \sqrt{\mu ^{2}-4\rho \sigma } \right) }{,}\\ \chi _{11}^{I}(\varrho )= \frac{2\sigma \sinh { \left( \sqrt{\mu ^{2}-4\rho \sigma }\varrho \right) }}{ \left( \sqrt{\mu ^{2}-4\rho \sigma }\cosh { \left( \sqrt{\mu ^{2}-4\rho \sigma }\varrho \right) }\pm \left( \sqrt{\mu ^{2}-4\rho \sigma } \right) \right) -\mu \sinh {\left( \sqrt{\mu ^{2}-4\rho \sigma }\varrho \right) }}{;} \end{array}\right. } \end{aligned}$$
    20 $$\begin{aligned}{} & {} {\left\{ \begin{array}{ll} \chi _{12}^{I}(\varrho )=\frac{4\sigma \cosh {\left( \frac{\sqrt{\mu ^{2}-4\rho \sigma }\varrho }{4} \right) }\sinh { \left( \frac{\sqrt{\mu ^{2}-4\rho \sigma }\varrho }{4} \right) }}{-2\mu \cosh {\left( \frac{\sqrt{\mu ^{2}-4\rho \sigma }\varrho }{4} \right) }\sinh {\left( \frac{\sqrt{\mu ^{2}-4\rho \sigma }\varrho }{4} \right) }+2\sqrt{\mu ^{2}-4\rho \sigma }\cosh ^{2}{\left( \frac{\sqrt{\mu ^{2}-4\rho \sigma }\varrho }{4} \right) }-\left( \sqrt{\mu ^{2}-4\rho \sigma } \right) }{,} \end{array}\right. } \end{aligned}$$
  - *Type 2* $$\mu ^{2}-4\rho \sigma <0$$, $$\mu \rho \ne 0$$ and $$\rho \sigma \ne 0$$:
    21 $$\begin{aligned}{} & {} {\left\{ \begin{array}{ll} \chi _{13}^{I}(\varrho )=\frac{-\mu +\sqrt{4\rho \sigma -\mu ^{2}} \tan { \left( \frac{\sqrt{4\rho \sigma -\mu ^{2}}\varrho }{2} \right) }}{2\rho }{,}\\ \chi _{14}^{I}(\varrho )=\frac{-\mu -\sqrt{4\rho \sigma -\mu ^{2}} \cot { \left( \frac{\sqrt{4\rho \sigma -\mu ^{2}}\varrho }{2} \right) }}{2\rho }{;} \end{array}\right. } \end{aligned}$$
    22 $$\begin{aligned}{} & {} {\left\{ \begin{array}{ll} \chi _{15}^{I}(\varrho )=\frac{-\mu +\sqrt{4\rho \sigma -\mu ^{2}} \left( \tan \left( {\sqrt{4\rho \sigma -\mu ^{2}}\varrho } \right) \pm \sec \left( {\sqrt{4\rho \sigma -\mu ^{2}}\varrho } \right) \right) }{2\rho }{,}\\ \chi _{16}^{I}(\varrho )=\frac{-\mu -\sqrt{4\rho \sigma -\mu ^{2}} \left( \cot \left( {\sqrt{4\rho \sigma -\mu ^{2}}\varrho } \right) \pm \csc \left( {\sqrt{4\rho \sigma -\mu ^{2}}\varrho } \right) \right) }{2\rho }{,}\\ \chi _{17}^{I}(\varrho )=\frac{-2\mu +\sqrt{4\rho \sigma -\mu ^{2}} \left( \tan { \left( \frac{\sqrt{4\rho \sigma -\mu ^{2}}}{4}\varrho \right) }-\cot {\left( \frac{\sqrt{4\rho \sigma -\mu ^{2}}}{4}\varrho \right) } \right) }{4\rho }{;} \end{array}\right. } \end{aligned}$$
    23 $$\begin{aligned}{} & {} {\left\{ \begin{array}{ll} \chi _{18}^{I}(\varrho )=\frac{1}{2\rho }(-\mu +\frac{\pm \sqrt{(E^{2}-F^{2})(4\rho \sigma -\mu ^{2})}-E(\sqrt{4\rho \sigma -\mu ^{2}})\cos (\sqrt{4\rho \sigma -\mu ^{2}}\varrho )}{E\sin (\sqrt{4\rho \sigma -\mu ^{2}}\varrho )+F}){,}\\ \chi _{19}^{I}(\varrho )=\frac{1}{2\rho }(-\mu -\frac{\pm \sqrt{(E^{2}-F^{2})(4\rho \sigma -\mu ^{2})}-E(\sqrt{4\rho \sigma -\mu ^{2}})\sin (\sqrt{4\rho \sigma -\mu ^{2}}\varrho )}{E\cos (\sqrt{4\rho \sigma -\mu ^{2}}\varrho )+F}){;} \end{array}\right. } \end{aligned}$$
    where E and F are non-zero constants that fulfill $E^{2}-F^{2}>0$.
    24 $$\begin{aligned}{} & {} {\left\{ \begin{array}{ll} \chi _{20}^{I}(\varrho )= \frac{2\sigma \cos { \left( \frac{\sqrt{4\rho \sigma -\mu ^{2}}}{2}\varrho \right) }}{\sqrt{4\rho \sigma -\mu ^{2}}\sin { \left( \frac{\sqrt{4\rho \sigma -\mu ^{2}}}{2}\varrho \right) }+\mu \cos { \left( \frac{\sqrt{4\rho \sigma -\mu ^{2}}}{2}\varrho \right) }}{,}\\ \chi _{21}^{I}(\varrho )= \frac{2\sigma \sin {\left( \frac{\sqrt{4\rho \sigma -\mu ^{2}}}{2}\varrho \right) }}{-\mu \sin {\left( \frac{\sqrt{4\rho \sigma -\mu ^{2}}}{2}\varrho \right) }+\sqrt{4\rho \sigma -\mu ^{2}}\cos {\left( \frac{\sqrt{4\rho \sigma -\mu ^{2}}}{2}\varrho \right) }}{;} \end{array}\right. } \end{aligned}$$
    25 $$\begin{aligned}{} & {} {\left\{ \begin{array}{ll} \left. \chi _{22}^{I}(\varrho )= - \frac{2\sigma \cos {\left( \sqrt{4\rho \sigma -\mu ^{2}}\varrho \right) }}{\sqrt{4\rho \sigma -\mu ^{2}}\sin { \left( \sqrt{4\rho \sigma -\mu ^{2}}\varrho \right) }+\mu \cos {\left( \sqrt{4\rho \sigma -\mu ^{2}}\varrho \right) } \pm \sqrt{4\rho \sigma -\mu ^{2}}} \right) {,}\\ \chi _{23}^{I}(\varrho )= \frac{2\sigma \sin {\left( \sqrt{4\rho \sigma -\mu ^{2}}\varrho \right) }}{\left( \sqrt{4\rho \sigma -\mu ^{2}}\cos {\left( \sqrt{4\rho \sigma -\mu ^{2}}\varrho \right) }\pm \left( \sqrt{4\rho \sigma -\mu ^{2}} \right) \right) -\mu \sin {\left( \sqrt{4\rho \sigma -\mu ^{2}}\varrho \right) }}{;} \end{array}\right. } \end{aligned}$$
    26 $$\begin{aligned}{} & {} {\left\{ \begin{array}{ll} \chi _{24}^{I}(\varrho )=\frac{4\sigma \cos {\left( \frac{\sqrt{4\rho \sigma -\mu ^{2}}\varrho }{4} \right) }\sin {\left( \frac{\sqrt{4\rho \sigma -\mu ^{2}}\varrho }{4} \right) }}{-2\mu \cos {\left( \frac{\sqrt{4\rho \sigma -\mu ^{2}}\varrho }{4} \right) }\sin {\left( \frac{\sqrt{4\rho \sigma -\mu ^{2}}\varrho }{4} \right) }+2\sqrt{\mu ^{2}-4\rho \sigma }\cos ^{2}{\left( \frac{\sqrt{4\rho \sigma -\mu ^{2}}\varrho }{4} \right) }-\left( \sqrt{4\rho \sigma -\mu ^{2}} \right) }{.} \end{array}\right. } \end{aligned}$$
- *Case 2*
  - *Type 1*$$\rho \sigma <0$$ and $$\rho \sigma \ne 0$$:
    27 $$\begin{aligned}{} & {} {\left\{ \begin{array}{ll} \chi _{1}^{II}(\varrho )=-\frac{\pm \sqrt{-2\rho \sigma }+\sqrt{-6\rho \sigma } \tanh { \left( \frac{\sqrt{-6\rho \sigma }\varrho }{2} \right) }}{2\rho }{,}\\ \chi _{2}^{II}(\varrho )=-\frac{\pm \sqrt{-2\rho \sigma }+\sqrt{-6\rho \sigma } \coth { \left( \frac{\sqrt{-6\rho \sigma }\varrho }{2} \right) }}{2\rho }{;} \end{array}\right. } \end{aligned}$$
    28 $$\begin{aligned}{} & {} {\left\{ \begin{array}{ll} \chi _{3}^{II}(\varrho )=-\frac{\pm \sqrt{-2\rho \sigma }+\sqrt{-6\rho \sigma }\left( \tanh \left( {\sqrt{-6\rho \sigma }\varrho } \right) \pm \iota \text {sech}\left( {\sqrt{-6\rho \sigma }\varrho } \right) \right) }{2\rho }{,}\\ \chi _{4}^{II}(\varrho )=-\frac{\pm \sqrt{-2\rho \sigma }+\sqrt{-6\rho \sigma } \left( \coth \left( {\sqrt{-6\rho \sigma }\varrho } \right) \pm \iota \text {csch} \left( {\sqrt{-6\rho \sigma }\varrho } \right) \right) }{2\rho }{,}\\ \chi _{5}^{II}(\varrho )=-\frac{\pm \sqrt{-2\rho \sigma }+\sqrt{-6\rho \sigma } \left( \tanh \left( \frac{\sqrt{-6\rho \sigma }\varrho }{4} \right) +\coth \left( \frac{\sqrt{-6\rho \sigma }\varrho }{4} \right) \right) }{4\rho }{;} \end{array}\right. } \end{aligned}$$
    29 $$\begin{aligned}{} & {} {\left\{ \begin{array}{ll} \chi _{6}^{II}(\varrho )=\frac{1}{2\rho } \left( \mp \sqrt{-2\rho \sigma }+\frac{\sqrt{ \left( E^{2}+F^{2} \right) (-6\rho \sigma )}-E\left( \sqrt{-6\rho \sigma } \right) \cosh \left( \sqrt{-6\rho \sigma }\varrho \right) }{E\sinh \left( \sqrt{-6\rho \sigma }\varrho \right) +F} \right) {,}\\ \chi _{7}^{II}(\varrho )=\frac{1}{2\rho } \left( \mp \sqrt{-2\rho \sigma }-\frac{\sqrt{ \left( F^{2}-E^{2} \right) \left( -6\rho \sigma \right) }+E \left( \sqrt{-6\rho \sigma } \right) \sinh \left( \sqrt{-6\rho \sigma }\varrho \right) }{E\cosh \left( \sqrt{-6\rho \sigma }\varrho \right) +F} \right) {;} \end{array}\right. } \end{aligned}$$
    where E and F are non-zero constants that fulfill $F^{2}-E^{2}>0$.
    30 $$\begin{aligned}{} & {} {\left\{ \begin{array}{ll} \chi _{8}^{II}(\varrho )= \frac{2\sigma \cosh { \left( \frac{\sqrt{-6\rho \sigma }\varrho }{2} \right) }}{\sqrt{-6\rho \sigma }\sinh { \left( \frac{\sqrt{-6\rho \sigma }\varrho }{2} \right) }\mp \sqrt{-2\rho \sigma }\cosh { \left( \frac{\sqrt{-6\rho \sigma }\varrho }{2} \right) }}{,}\\ \chi _{9}^{II}(\varrho )= \frac{-2\sigma \sinh { \left( \frac{\sqrt{-6\rho \sigma }\varrho }{2} \right) }}{\pm \sqrt{-2\rho \sigma }\sinh {\left( \frac{\sqrt{-6\rho \sigma }\varrho }{2} \right) }-\sqrt{-6\rho \sigma }\cosh {\left( \frac{\sqrt{-6\rho \sigma }\varrho }{2} \right) }}{;} \end{array}\right. } \end{aligned}$$
    31 $$\begin{aligned}{} & {} {\left\{ \begin{array}{ll} \left. \chi _{10}^{II}(\varrho )= \frac{2\sigma \cosh {\left( \sqrt{-6\rho \sigma }\varrho \right) }}{\sqrt{-6\rho \sigma }\sinh {\left( \sqrt{-6\rho \sigma }\varrho \right) }\mp \sqrt{-2\rho \sigma }\cosh {\left( \sqrt{-6\rho \sigma }\varrho \right) }\pm \iota \sqrt{-6\rho \sigma } } \right) {,}\\ \chi _{11}^{II}(\varrho )= \frac{2\sigma \sinh {(\sqrt{-6\rho \sigma }\varrho )}}{\mp \sqrt{-2\rho \sigma }\sinh {(\sqrt{-6\rho \sigma }\varrho )}+\sqrt{-6\rho \sigma }\cosh {(\sqrt{-6\rho \sigma }\varrho )}\pm \iota \sqrt{-6\rho \sigma })}{;} \end{array}\right. } \end{aligned}$$
    32 $$\begin{aligned}{} & {} {\left\{ \begin{array}{ll} \chi _{12}^{II}(\varrho )=\frac{4\sigma \cosh {(\frac{\sqrt{-6\rho \sigma }\varrho }{4})}\sinh {(\frac{\sqrt{-6\rho \sigma }\varrho }{4})}}{\pm 2\sqrt{-2\rho \sigma }\cosh {(\frac{\sqrt{-6\rho \sigma }\varrho }{4})}\sinh {(\frac{\sqrt{-6\rho \sigma }\varrho }{4})}+2\sqrt{-6\rho \sigma }\cosh ^{2}{(\frac{\sqrt{-6\rho \sigma }\varrho }{4})}-(\sqrt{-6\rho \sigma })}{.} \end{array}\right. } \end{aligned}$$
- *Case 3*
  - *Type 1* When $c_{2}=1, c_{3}=-\frac{2\alpha _{3}}{\alpha _{1}}, c_{4}=\frac{\alpha _{3}^{2}-\alpha _{2}^{2}}{\alpha _{1}^{2}}$, listed below is the solution to Eq. (11)
    33 $$\begin{aligned} {\left\{ \begin{array}{ll} \chi _{1}^{III}(\varrho )=\frac{\alpha _{1}sech(\varrho )}{\alpha _{2}+\alpha _{3}sech(\varrho )}{.} \end{array}\right. } \end{aligned}$$
  - *Type 2* When $c_{2}=1, c_{3}=-\frac{2\alpha _{3}}{\alpha _{1}}, c_{4}=\frac{\alpha _{3}^{2}+\alpha _{2}^{2}}{\alpha _{1}^{2}}$, listed below is the solution to Eq. (11)
    34 $$\begin{aligned} {\left\{ \begin{array}{ll} \chi _{2}^{III}(\varrho )=\frac{\alpha _{1}csch(\varrho )}{\alpha _{2}+\alpha _{3}csch(\varrho )}{.} \end{array}\right. } \end{aligned}$$
  - *Type 3* When $c_{2}=4, c_{3}=-\frac{4(2\alpha _{2}+\alpha _{4})}{\alpha _{1}}, c_{4}=\frac{\alpha _{3}^{2}+4\alpha _{2}^{2}+4\alpha _{2}\alpha _{4}}{\alpha _{1}^{2}}$, listed below is the solution to Eq. (11)
    35 $$\begin{aligned} {\left\{ \begin{array}{ll} \chi _{3}^{III}(\varrho )=\frac{\alpha _{1}sech^{2}(\varrho )}{\alpha _{2}sech^{2}(\varrho )+\alpha _{3}\tanh (\varrho )+\alpha _{4}}{.} \end{array}\right. } \end{aligned}$$
  - *Type 4* When $c_{2}=4, c_{3}=-\frac{4(\alpha _{4}-2\alpha _{2})}{\alpha _{1}}, c_{4}=\frac{\alpha _{3}^{2}+4\alpha _{2}^{2}-4\alpha _{2}\alpha _{4}}{\alpha _{1}^{2}}$, listed below is the solution to Eq. (11)
    36 $$\begin{aligned} {\left\{ \begin{array}{ll} \chi _{4}^{III}(\varrho )=\frac{\alpha _{1}csch^{2}(\varrho )}{\alpha _{2}\coth ^{2}(\varrho )+\alpha _{3}\tanh (\varrho )+\alpha _{4}}{.} \end{array}\right. } \end{aligned}$$
  - *Type 5* When $c_{2}=\alpha _{1}^{2}, c_{3}=2\alpha _{1}\alpha _{2}, c_{4}=\alpha _{2}^{2}$, listed below is the solution to Eq. (11)
    37 $$\begin{aligned} {\left\{ \begin{array}{ll} \chi _{5}^{III}(\varrho )=-\frac{\alpha _{1}\alpha _{3}}{\alpha _{2}(\cosh (\alpha _{1}\varrho )-\sinh (\alpha _{1}\varrho )+\alpha _{3})}{,}\\ \chi _{6}^{III}(\varrho )=-\frac{\alpha _{1}(\sinh (\alpha _{1}\varrho )+\cosh (\alpha _{1}\varrho ))}{\alpha _{2}(\sinh (\alpha _{1}\varrho )+\cosh (\alpha _{1}\varrho )+\alpha _{3})}{.} \end{array}\right. } \end{aligned}$$
  - *Type 6* When $c_{2}=-1, c_{3}=\frac{2\alpha _{3}}{\alpha _{1}}, c_{4}=-\frac{\alpha _{3}^{2}-\alpha _{2}^{2}}{\alpha _{1}^{2}}$, listed below is the solution to Eq. (11)
    38 $$\begin{aligned} {\left\{ \begin{array}{ll} \chi _{7}^{III}(\varrho )=\frac{\alpha _{1}\sec (\varrho )}{\alpha _{2}+\alpha _{3}\sec (\varrho )}{,}\\ \chi _{8}^{III}(\varrho )=\frac{\alpha _{1}\csc (\varrho )}{\alpha _{2}+\alpha _{3}\csc (\varrho )}{.} \end{array}\right. } \end{aligned}$$
  - *Type 7* When $c_{2}=-4, c_{3}=\frac{4(2\alpha _{2}+\alpha _{4})}{\alpha _{1}}, c_{4}=-\frac{-\alpha _{3}^{2}+4\alpha _{2}^{2}+4\alpha _{2}\alpha _{4}}{\alpha _{1}^{2}}$, listed below is the solution to Eq. (11)
    39 $$\begin{aligned} {\left\{ \begin{array}{ll} \chi _{9}^{III}(\varrho )=\frac{\alpha _{1}\sec ^{2}(\varrho )}{\alpha _{2}\sec ^{2}(\varrho )+\alpha _{3}\tan (\varrho )+\alpha _{4}}{,}\\ \chi _{10}^{III}(\varrho )=\frac{\alpha _{1}\csc ^{2}(\varrho )}{\alpha _{2}\csc ^{2}(\varrho )+\alpha _{3}\cot (\varrho )+\alpha _{4}}{.} \end{array}\right. } \end{aligned}$$
    where the constants $\alpha _{1}, \alpha _{2}, \alpha _{3}$ and $\alpha _{4}$ are arbitrary.
- *Case 4* In the present scenario, the common solutions to Eq. (12) are the single and combination non-degenerative Jacobi elliptic functions (JEFs). The association among the parameters of $c_{0}, c_{2}$ and $c_{4}$ in regard of solution of JEF given in NLODE Eq. (12), are depicted in a subsequent Table 1. Tables 1, 2, and 3 listed below, accordingly, describe the categories.
- *Case 5* By way of illustration, in the case where Eq. (14) is gruntled the answer is Weierstrass elliptic doubly periodic type solution.
  40 $$\begin{aligned} \chi _{1}^{V}(\varrho )=\digamma \left( \frac{\sqrt{c_{3}}}{2}\varrho , \frac{-4c_{1}}{c_{3}}, \frac{-4c_{0}}{c_{3}} \right) \ \ \ \ \ \ c_{3}>0. \end{aligned}$$

**Table 1:** The association among the parameters of $c_{0}, c_{2}$ and $c_{4}$ in regard of solution of JEF given in Eq. (12) where $0\le q\le 1$.

$c_{0}$	$c_{2}$	$c_{4}$	$\chi _{l}^{IV}(\varrho )$
1	$-(1-q^{2})$	$q^{2}$	$\chi _{1}^{IV}(\varrho )=cn\varrho , \chi _{2}^{IV}(\varrho )=cd\varrho =\frac{cn\varrho }{cd\varrho }$
$1-q^{2}$	$2q^{2}-1$	$-q^{2}$	$\chi _{3}^{IV}(\varrho )=cn\varrho$
$q^{2}-1$	$2-q^{2}$	$-1$	$\chi _{4}^{IV}(\varrho )=dn\varrho$
$q^{2}$	$-(1+q^{2})$	1	$\chi _{5}^{IV}(\varrho )=nr\varrho =(rn\varrho )^{-1}, \chi _{6}^{IV}(\varrho )=dc\varrho =\frac{dn\varrho }{cn\varrho }$
$-q^{2}$	$2q^{2}-1$	$1-q^{2}$	$\chi _{7}^{IV}(\varrho )=nc\varrho =(cn\varrho )^{-1}$
$-1$	$2-q^{2}$	$q^{2}-1$	$\chi _{8}^{IV}(\varrho )=nd\varrho =(dn\varrho )^{-1}$
1	$2-q^{2}$	$1-q^{2}$	$\chi _{9}^{IV}(\varrho )=rc\varrho =\frac{rn\varrho }{cn\varrho }$
1	$2q^{2}-1$	$-q^{2}(1-q^{2})$	$\chi _{10}^{IV}(\varrho )=rd\varrho =\frac{rn\varrho }{dn\varrho }$
$1-q^{2}$	$2-q^{2}$	1	$\chi _{11}^{IV}(\varrho )=cr\varrho =\frac{cn\varrho }{rn\varrho }$
$-q^{2}(1-q^{2})$	$2q^{2}-1$	1	$\chi _{12}^{IV}(\varrho )=dr\varrho =\frac{dn\varrho }{rn\varrho }$
$\frac{1}{4}$	$\frac{1-2q^{2}}{2}$	*frac*14	$\chi _{13}^{IV}(\varrho )=nr\varrho \pm cr\varrho$
$\frac{1-q^{2}}{4}$	$\frac{1+q^{2}}{2}$	$\frac{1-q^{2}}{4}$	$\chi _{14}^{IV}(\varrho )=nc\varrho \pm rc\varrho$
$\frac{q^{2}}{4}$	$\frac{q^{2}-2}{2}$	$\frac{1}{4}$	$\chi _{15}^{IV}(\varrho )=nr\varrho \pm dr\varrho$
$\frac{q^{2}}{4}$	$\frac{q^{2}-2}{2}$	$\frac{q^{2}}{4}$	$\chi _{16}^{IV}(\varrho )=rn\varrho \pm \iota cr\varrho$

**Table 2:** The JEFs become hyperbolic functions if $q\rightarrow 1$.

JEF	HF	JEF	HF
$rn \, \varrho$	$\tanh \, \varrho$	$nr \, \varrho$	$\coth \, \varrho$
$cn \, \varrho$	$sech \, \varrho$	$nc \, \varrho$	$\cosh \, \varrho$
$dn \, \varrho$	$sech \, \varrho$	$nd \, \varrho$	$\cosh \, \varrho$
$rc \, \varrho$	$\sinh \, \varrho$	$cr \, \varrho$	$csch \, \varrho$
$rd \, \varrho$	$\sinh \, \varrho$	$dr \, \varrho$	$csch \, \varrho$
$cd \, \varrho$	1	$dc \, \varrho$	1

**Table 3:** The JEFs become trigonometric functions if $q\rightarrow 0$.

JEF	HF	JEF	HF
$rn \, \varrho$	$\sin \, \varrho$	$nr \, \varrho$	$\csc \, \varrho$
$cn \, \varrho$	$\cos \, \varrho$	$nc \, \varrho$	$\sec \, \varrho$
$dn \, \varrho$	1	$nd \, \varrho$	1
$rc \, \varrho$	$\tan \, \varrho$	$cr \, \varrho$	$\cot \, \varrho$
$rd \, \varrho$	$\sin \, \varrho$	$dr \, \varrho$	$\csc \, \varrho$
$cd \, \varrho$	$\cos \, \varrho$	$dc \, \varrho$	$\sec \, \varrho$

**Figure 1:**
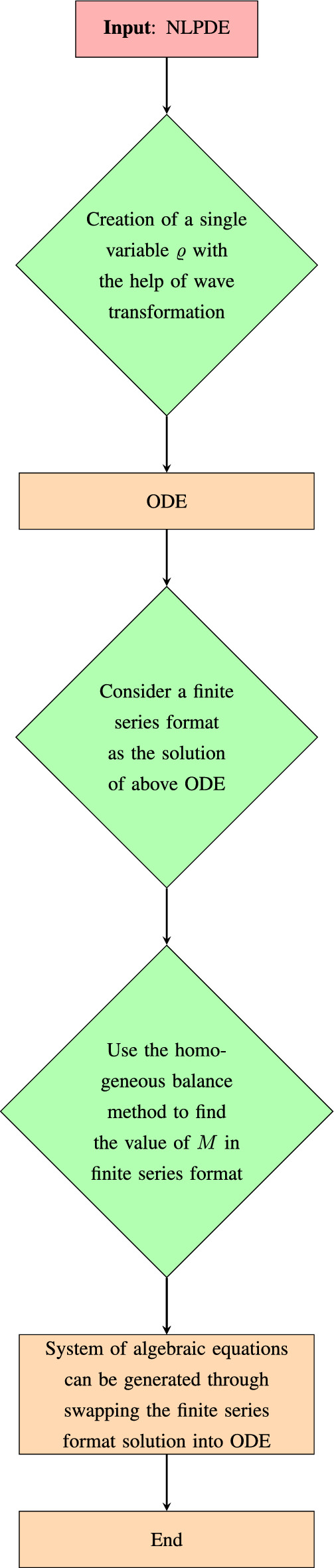
Algorithm flowchart of the MEFSEM.

## Mathematical formulation and implementation of the MEFSEM

In current portion, A D-CDNA model fabricated from elastic rods is laid out. NL equations demonstrate the dynamics of this model, that can be expressed as

41 $$\begin{aligned} \Theta _{tt}-\Lambda _{1}^{2}\Theta _{ss}= & {} \Lambda _{2}\Theta +\Lambda _{3}\Theta \Xi +\Lambda _{4}\Theta ^{3}+\Lambda _{5}\Theta \Xi ^{2}{,} \end{aligned}$$

42 $$\begin{aligned} \Xi _{tt}-\Lambda _{6}^{2}\Xi _{ss}= & {} \Lambda _{7}\Xi +\Lambda _{8}\Theta ^{2}+\Lambda _{9}\Theta ^{2}\Xi +\Lambda _{10}\Xi ^{3}+\Lambda _{11}{,} \end{aligned}$$

where the function $\Theta$, reflects the disparity between the longitudinal displacements of the foremost and bottom conductors in Eqs. (41) and (42), whereas the variation between the highest and lowest strands’ transverse displacements is represented by $\Xi$. The constants $\Lambda _{1}, \Lambda _{2}, \ldots \Lambda _{11}$ are written as

43 $$\begin{aligned} \Lambda _{1}= & {} \pm \frac{Z}{\delta }, \Lambda _{2}=\pm \frac{-2\omega (y-a_{0})}{\delta \Gamma h}, \Lambda _{3}=2\Lambda _{8}=\frac{2\sqrt{2}\omega a_{0}}{\Delta \Gamma h^{2}},\nonumber \\ \Lambda _{4}= & {} \Lambda _{9}=\frac{-2\omega a_{0}}{\Delta \Gamma h^{2}}, \Lambda _{5}=\Lambda _{10}=\frac{4\omega a_{0}}{\Delta \Gamma h^{3}}, \Lambda _{6}=\pm \frac{g}{\Delta }, \Lambda _{11}=\frac{\sqrt{2}\omega (h-a_{0})}{\Delta \Gamma }, \end{aligned}$$

where, $a_{0}, h, \omega , g, Z, \Gamma$ and $\Delta$ demonstrate the membrane’s height, separation of the two strands, the elastic membrane’s rigidity, density of stress in every thread, Young modulus, cross-sectional area and mass density respectively.

Assume the subsequent transformation

44 $$\begin{aligned} \Xi = b_{0}\Theta +b_{1}{,} \end{aligned}$$

where $b_{0}$ and $b_{1}$ are constants. Equations (41) and (42) are streamlined into the two subsequent forms because of (44).

45 $$\begin{aligned}{} & {} \Theta _{tt}-\Lambda _{1}^{2}\Theta _{ss}= \Theta ^{3}(\Lambda _{4}+\Lambda _{5}b_{0}^{2})+\Theta ^{2}(2\Lambda _{5}b_{0}b_{1}+\Lambda _{3}b_{0})+\Theta (\Lambda _{2}+b_{1}\Lambda _{3}+\Lambda _{5}b_{1}^{2}), \end{aligned}$$

46 $$\begin{aligned}{} & {} \Theta _{tt}-\Lambda _{6}^{2}\Theta _{ss}= \Theta ^{3}(\Lambda _{9}+\Lambda _{10}b_{0}^{2})+\Theta ^{2}(3\Lambda _{10}b_{0}b_{1}+\frac{\Lambda _{8}}{b_{0}}+\frac{\Lambda _{9}b_{1}}{b_{0}})+\Theta (\Lambda _{7}+3\Lambda _{10}b_{1}^{2})+\frac{\Lambda _{7}b_{1}}{b_{0}}+\frac{\Lambda _{10}b_{1}^{3}}{b_{0}}+\frac{\Lambda _{11}}{b_{0}}. \end{aligned}$$

Equations (45) and (46) can be compared with Eq. (44) to determine that $b_{1}=\frac{h}{\sqrt{2}}$ and $Z=g$. At this point, Eqs. (45) and (46) can be expressed as

47 $$\begin{aligned} \Theta _{tt}-\Lambda _{1}^{2}\Theta _{ss}= \Phi _{1}\Theta ^{3}+\Phi _{2}\Theta ^{2}+\Phi _{3}\Theta , \end{aligned}$$

where $$\Phi _{1}=\frac{\kappa (-2+4b_{0}^{2})}{h^{3}}, \Phi _{2}= \frac{6\sqrt{2}b_{0}\kappa }{h^{2}}, \Phi _{3}=\frac{-2\kappa }{a_{0}}+\frac{6\kappa }{h}, \kappa = \frac{\omega a_{0}}{\Delta \Gamma }$$ and $$\Lambda _{1}=\pm \frac{Z}{\delta }$$.

Applying the wave transformations specified below

48 $$\begin{aligned} \Theta (s, t) = \Upsilon (\varrho ),\ \ \ \ \ \varrho = \xi s + \zeta t{.} \end{aligned}$$

Equation (47) reduce to the listed below system of ODE as a result of replacing Eq. (48) into Eq. (47)

49 $$\begin{aligned} (\zeta ^{2}-\xi ^{2}\Lambda _{1}^{2})\Upsilon ''-\Phi _{1}\Upsilon ^{3}-\Phi _{2}\Upsilon ^{2}-\Phi _{3}\Upsilon =0, \ \ \ \ \ \zeta ^{2}-\xi ^{2}\Lambda _{1}^{2}\ne 0{,} \end{aligned}$$

where prime stands for derivative with respect to $\varrho$. Employing the homogeneous balancing technique on the highest order derivative linear term $\Upsilon ''$ and the highest power non-linear term $\Upsilon ^3$ given in Eq. (49) provides $3M = M + 2$ which gives $M = 1$.

Accordingly, Eq. (4) translates to

50 $$\begin{aligned} \Upsilon (\varrho ) = \eta _{0}+\eta _{1}\chi (\varrho ){.} \end{aligned}$$

The polynomial equation in the shape of $\chi (\varrho )$ for Eq. (49) can be uncovered in the following manner with the help of the Eqs. (50) and (5)

51 $$\begin{aligned}{} & {} \left( 4\zeta ^{2}c_{4}\eta _{1}-4\xi ^{2}c_{4}\eta _{1}\Lambda _{1}^{2}-2\eta _{1}^{3}\Phi _{1} \right) (\chi (\varrho ))^3+ \left( 3\zeta ^{2}c_{3}\eta _{1}-3\xi ^{2}c_{3}\eta _{1}\Lambda _{1}^{2}-6\eta _{0}\eta _{1}^{2}\Phi _{1}-2\eta _{1}^{2}\Phi _{2} \right) (\chi (\varrho ))^2\nonumber \\{} & {} \quad + \left( 2\zeta ^{2}c_{2}\eta _{1}-2\xi ^{2}c_{2}\eta _{1}\Lambda _{1}^{2}-2\eta _{1}\Phi _{3}-6\eta _{0}^{2}\eta _{1}\Phi _{1}-4\eta _{0}\eta _{1}\Phi _{2} \right) (\chi (\varrho ))\nonumber \\{} & {} \quad +\zeta ^{2}c_{1}\eta _{1}-\xi ^{2}c_{1}\eta _{1}\Lambda _{1}^{2}-2\eta _{0}^{3}\Phi _{1}-2\eta _{0}^{2}\Phi _{2}-2\eta _{0}\Phi _{3}=0{.} \end{aligned}$$

In order to get the set of algebraic equations, the coefficient of comparable power of $\chi (\varrho )$ in Eq. (51) equal to zero.

$$\begin{aligned} {\left\{ \begin{array}{ll} (\chi (\varrho ))^3: 4\zeta ^{2}c_{4}\eta _{1}-4\xi ^{2}c_{4}\eta _{1}\Lambda _{1}^{2}-2\eta _{1}^{3}\Phi _{1} = 0,\\ (\chi (\varrho ))^2: 3\zeta ^{2}c_{3}\eta _{1}-3\xi ^{2}c_{3}\eta _{1}\Lambda _{1}^{2}-6\eta _{0}\eta _{1}^{2}\Phi _{1}-2\eta _{1}^{2}\Phi _{2} = 0,\\ (\chi (\varrho ))^1: 2\zeta ^{2}c_{2}\eta _{1}-2\xi ^{2}c_{2}\eta _{1}\Lambda _{1}^{2}-2\eta _{1}\Phi _{3}-6\eta _{0}^{2}\eta _{1}\Phi _{1}-4\eta _{0}\eta _{1}\Phi _{2} = 0,\\ (\chi (\varrho ))^0: \zeta ^{2}c_{1}\eta _{1}-\xi ^{2}c_{1}\eta _{1}\Lambda _{1}^{2}-2\eta _{0}^{3}\Phi _{1}-2\eta _{0}^{2}\Phi _{2}-2\eta _{0}\Phi _{3} = 0. \end{array}\right. } \end{aligned}$$

Solve above system of algebraic equations with the aid of Mathematica 13.2 gives

52 $$\begin{aligned} {\eta _0} = \frac{3\zeta ^{2}c_{3}-3\xi ^{2}c_{3}\Lambda _{1}^{2}-2\eta _{1}\Phi _{2}}{6\eta _{1}\Phi _{1}}, {\eta _1} = \mp \sqrt{\frac{2(\zeta ^{2}c_{4}-\xi ^{2}c_{4}\Lambda _{1}^{2})}{\Phi _{1}}}. \end{aligned}$$

The TW solutions of Eqs. (41)–(42) that result from replacing Eqs. (52) and (15)–(40) into Eq. (50) are formulated as follows:

### Special cases

### Case 1

#### Type 1: $\mu ^{2}-4\rho \sigma >0$, $\mu \rho \ne 0$, $\rho \sigma \ne 0$

53 $$\begin{aligned} \Theta _{1,1,1}(s, t)= & {} \frac{3\zeta ^{2}c_{3}-3\xi ^{2}c_{3}\Lambda _{1}^{2}-2\eta _{1}\Phi _{2}}{6\eta _{1}\Phi _{1}}\mp \sqrt{\frac{2 \left( \zeta ^{2}c_{4}-\xi ^{2}c_{4}\Lambda _{1}^{2} \right) }{\Phi _{1}}}\nonumber \\{} & {} \left( -\frac{\mu +\sqrt{\mu ^{2}-4\rho \sigma } \left( \tanh \left( {\sqrt{\mu ^{2}-4\rho \sigma }\varrho } \right) \pm \iota \text {sech} \left( {\sqrt{\mu ^{2}-4\rho \sigma }\varrho } \right) \right) }{2\rho } \right) {,} \end{aligned}$$

54 $$\begin{aligned} \Theta _{1,1,2}(s, t)= & {} \frac{3\zeta ^{2}c_{3}-3\xi ^{2}c_{3}\Lambda _{1}^{2}-2\eta _{1}\Phi _{2}}{6\eta _{1}\Phi _{1}}\mp \sqrt{\frac{2\left( \zeta ^{2}c_{4}-\xi ^{2}c_{4}\Lambda _{1}^{2} \right) }{\Phi _{1}}}\nonumber \\{} & {} \left( -\frac{\mu +\sqrt{\mu ^{2}-4\rho \sigma } \coth { \left( \frac{\sqrt{\mu ^{2}-4\rho \sigma }\varrho }{2} \right) }}{2\rho } \right) {,} \end{aligned}$$

55 $$\begin{aligned} \Theta _{1,1,3}(s, t)= & {} \frac{3\zeta ^{2}c_{3}-3\xi ^{2}c_{3}\Lambda _{1}^{2}-2\eta _{1}\Phi _{2}}{6\eta _{1}\Phi _{1}}\mp \sqrt{\frac{2\left( \zeta ^{2}c_{4}-\xi ^{2}c_{4}\Lambda _{1}^{2} \right) }{\Phi _{1}}}\nonumber \\{} & {} \left( -\frac{\mu +\sqrt{\mu ^{2}-4\rho \sigma } \left( \tanh \left( {\sqrt{\mu ^{2}-4\rho \sigma }\varrho } \right) \pm \iota \text {sech}\left( {\sqrt{\mu ^{2}-4\rho \sigma }\varrho } \right) \right) }{2\rho } \right) {,} \end{aligned}$$

56 $$\begin{aligned} \Theta _{1,1,4}(s, t)= & {} \frac{3\zeta ^{2}c_{3}-3\xi ^{2}c_{3}\Lambda _{1}^{2}-2\eta _{1}\Phi _{2}}{6\eta _{1}\Phi _{1}}\mp \sqrt{\frac{2\left( \zeta ^{2}c_{4}-\xi ^{2}c_{4}\Lambda _{1}^{2} \right) }{\Phi _{1}}}\nonumber \\{} & {} \left( -\frac{\mu +\sqrt{\mu ^{2}-4\rho \sigma } \left( \coth \left( {\sqrt{\mu ^{2}-4\rho \sigma }\varrho } \right) \pm \text {csch} \left( {\sqrt{\mu ^{2}-4\rho \sigma }\varrho } \right) \right) }{2\rho } \right) {,} \end{aligned}$$

57 $$\begin{aligned} \Theta _{1,1,5}(s, t)= & {} \frac{3\zeta ^{2}c_{3}-3\xi ^{2}c_{3}\Lambda _{1}^{2}-2\eta _{1}\Phi _{2}}{6\eta _{1}\Phi _{1}}\mp \sqrt{\frac{2\left( \zeta ^{2}c_{4}-\xi ^{2}c_{4}\Lambda _{1}^{2} \right) }{\Phi _{1}}}\nonumber \\{} & {} \left( -\frac{2\mu +\sqrt{\mu ^{2}-4\rho \sigma } \left( \tanh { \left( \frac{\sqrt{\mu ^{2}-4\rho \sigma }}{4}\varrho \right) }+\coth { \left( \frac{\sqrt{\mu ^{2}-4\rho \sigma }}{4}\varrho \right) } \right) }{4\rho } \right) {,} \end{aligned}$$

58 $$\begin{aligned} \Theta _{1,1,6}(s, t)= & {} \frac{3\zeta ^{2}c_{3}-3\xi ^{2}c_{3}\Lambda _{1}^{2}-2\eta _{1}\Phi _{2}}{6\eta _{1}\Phi _{1}}\mp \sqrt{\frac{2 \left( \zeta ^{2}c_{4}-\xi ^{2}c_{4}\Lambda _{1}^{2}\right) }{\Phi _{1}}}\nonumber \\{} & {} \left( \frac{1}{2\rho }\left( -\mu +\frac{\sqrt{\left( E^{2}+F^{2}\right) \left( \mu ^{2}-4\rho \sigma \right) }-E\left( \sqrt{\mu ^{2}-4\rho \sigma }\right) \cosh \left( \sqrt{\mu ^{2}-4\rho \sigma }\varrho \right) }{E\sinh \left( \sqrt{\mu ^{2}-4\rho \sigma }\varrho \right) +F}\right) \right) {,} \end{aligned}$$

59 $$\begin{aligned} \Theta _{1,1,7}(s, t)= & {} \frac{3\zeta ^{2}c_{3}-3\xi ^{2}c_{3}\Lambda _{1}^{2}-2\eta _{1}\Phi _{2}}{6\eta _{1}\Phi _{1}}\mp \sqrt{\frac{2\left( \zeta ^{2}c_{4}-\xi ^{2}c_{4}\Lambda _{1}^{2} \right) }{\Phi _{1}}}\nonumber \\{} & {} \left( \frac{1}{2\rho }\left( -\mu -\frac{\sqrt{\left( F^{2}-E^{2}\right) \left( \mu ^{2}-4\rho \sigma \right) }+E\left( \sqrt{\mu ^{2}-4\rho \sigma }\right) \sinh \left( \sqrt{\mu ^{2}-4\rho \sigma }\varrho \right) }{E\cosh \left( \sqrt{\mu ^{2}-4\rho \sigma }\varrho \right) +F}\right) \right) {,} \end{aligned}$$

60 $$\begin{aligned} \Theta _{1,1,8}(s, t)= & {} \frac{3\zeta ^{2}c_{3}-3\xi ^{2}c_{3}\Lambda _{1}^{2}-2\eta _{1}\Phi _{2}}{6\eta _{1}\Phi _{1}}\mp \sqrt{\frac{2 \left( \zeta ^{2}c_{4}-\xi ^{2}c_{4}\Lambda _{1}^{2}\right) }{\Phi _{1}}}\nonumber \\{} & {} \left( \frac{2\sigma \cosh {\left( \frac{\sqrt{\mu ^{2}-4\rho \sigma }\varrho }{2}\right) }}{\sqrt{\mu ^{2}-4\rho \sigma }\sinh {\left( \frac{\sqrt{\mu ^{2}-4\rho \sigma }\varrho }{2}\right) }-\mu \cosh {\left( \frac{\sqrt{\mu ^{2}-4\rho \sigma }\varrho }{2}\right) }}\right) {,} \end{aligned}$$

61 $$\begin{aligned} \Theta _{1,1,9}(s, t)= & {} \frac{3\zeta ^{2}c_{3}-3\xi ^{2}c_{3}\Lambda _{1}^{2}-2\eta _{1}\Phi _{2}}{6\eta _{1}\Phi _{1}}\mp \sqrt{\frac{2\left( \zeta ^{2}c_{4}-\xi ^{2}c_{4}\Lambda _{1}^{2} \right) }{\Phi _{1}}} \nonumber \\{} & {} \left( \frac{-2\sigma \sinh {\left( \frac{\sqrt{\mu ^{2}-4\rho \sigma }\varrho }{2}\right) }}{\mu \sinh {\left( \frac{\sqrt{\mu ^{2}-4\rho \sigma }\varrho }{2}\right) }-\sqrt{\mu ^{2}-4\rho \sigma }\cosh {\left( \frac{\sqrt{\mu ^{2}-4\rho \sigma }\varrho }{2}\right) }}\right) {,} \end{aligned}$$

62 $$\begin{aligned} \Theta _{1,1,10}(s, t)= & {} \frac{3\zeta ^{2}c_{3}-3\xi ^{2}c_{3}\Lambda _{1}^{2}-2\eta _{1}\Phi _{2}}{6\eta _{1}\Phi _{1}}\mp \sqrt{\frac{2\left( \zeta ^{2}c_{4}-\xi ^{2}c_{4}\Lambda _{1}^{2}\right) }{\Phi _{1}}}\nonumber \\{} & {} \left( \frac{2\sigma \cosh {\left( \sqrt{\mu ^{2}-4\rho \sigma }\varrho \right) }}{\sqrt{\mu ^{2}-4\rho \sigma }\sinh {\left( \sqrt{\mu ^{2}-4\rho \sigma }\varrho \right) }-\left( \mu \cosh {\left( \sqrt{\mu ^{2}-4\rho \sigma }\varrho \right) }\pm \iota \sqrt{\mu ^{2}-4\rho \sigma }\right) }\right) {,} \end{aligned}$$

63 $$\begin{aligned} \Theta _{1,1,11}(s, t)= & {} \frac{3\zeta ^{2}c_{3}-3\xi ^{2}c_{3}\Lambda _{1}^{2}-2\eta _{1}\Phi _{2}}{6\eta _{1}\Phi _{1}}\mp \sqrt{\frac{2\left( \zeta ^{2}c_{4}-\xi ^{2}c_{4}\Lambda _{1}^{2}\right) }{\Phi _{1}}}\nonumber \\{} & {} \left( \frac{2\sigma \sinh {\left( \sqrt{\mu ^{2}-4\rho \sigma }\varrho \right) }}{\left( \sqrt{\mu ^{2}-4\rho \sigma }\cosh {\left( \sqrt{\mu ^{2}-4\rho \sigma }\varrho \right) }\pm \left( \sqrt{\mu ^{2}-4\rho \sigma }\right) \right) -\mu \sinh {\left( \sqrt{\mu ^{2}-4\rho \sigma }\varrho \right) }}\right) {,} \end{aligned}$$

64 $$\begin{aligned} \Theta _{1,1,12}(s, t)= & {} \frac{3\zeta ^{2}c_{3}-3\xi ^{2}c_{3}\Lambda _{1}^{2}-2\eta _{1}\Phi _{2}}{6\eta _{1}\Phi _{1}}\mp \sqrt{\frac{2\left( \zeta ^{2}c_{4}-\xi ^{2}c_{4}\Lambda _{1}^{2}\right) }{\Phi _{1}}}\nonumber \\{} & {} \left( \frac{4\sigma \cosh {\left( \frac{\sqrt{\mu ^{2}-4\rho \sigma }\varrho }{4}\right) }\sinh {\left( \frac{\sqrt{\mu ^{2}-4\rho \sigma }\varrho }{4}\right) }}{-2\mu \cosh {\left( \frac{\sqrt{\mu ^{2}-4\rho \sigma }\varrho }{4}\right) }\sinh {\left( \frac{\sqrt{\mu ^{2}-4\rho \sigma }\varrho }{4}\right) }+2\sqrt{\mu ^{2}-4\rho \sigma }\cosh ^{2}{\left( \frac{\sqrt{\mu ^{2}-4\rho \sigma }\varrho }{4}\right) }-\left( \sqrt{\mu ^{2}-4\rho \sigma }\right) }\right) {.} \end{aligned}$$

#### Type 2: $\mu ^{2}-4\rho \sigma <0$, $\mu \rho \ne 0$, $\rho \sigma \ne 0$

65 $$\begin{aligned} \Theta _{1,2,13}(s, t)= & {} \frac{3\zeta ^{2}c_{3}-3\xi ^{2}c_{3}\Lambda _{1}^{2}-2\eta _{1}\Phi _{2}}{6\eta _{1}\Phi _{1}}\mp \sqrt{\frac{2 \left( \zeta ^{2}c_{4}-\xi ^{2}c_{4}\Lambda _{1}^{2}\right) }{\Phi _{1}}}\left( \frac{-\mu +\sqrt{4\rho \sigma -\mu ^{2}} \tan {\left( \frac{\sqrt{4\rho \sigma -\mu ^{2}}\varrho }{2}\right) }}{2\rho }\right) {,} \end{aligned}$$

66 $$\begin{aligned} \Theta _{1,2,14}(s, t)= & {} \frac{3\zeta ^{2}c_{3}-3\xi ^{2}c_{3}\Lambda _{1}^{2}-2\eta _{1}\Phi _{2}}{6\eta _{1}\Phi _{1}}\mp \sqrt{\frac{2\left( \zeta ^{2}c_{4}-\xi ^{2}c_{4}\Lambda _{1}^{2}\right) }{\Phi _{1}}}\left( \frac{-\mu -\sqrt{4\rho \sigma -\mu ^{2}} \cot {\left( \frac{\sqrt{4\rho \sigma -\mu ^{2}}\varrho }{2}\right) }}{2\rho }\right) {,} \end{aligned}$$

67 $$\begin{aligned} \Theta _{1,2,15}(s, t)= & {} \frac{3\zeta ^{2}c_{3}-3\xi ^{2}c_{3}\Lambda _{1}^{2}-2\eta _{1}\Phi _{2}}{6\eta _{1}\Phi _{1}}\mp \sqrt{\frac{2\left( \zeta ^{2}c_{4}-\xi ^{2}c_{4}\Lambda _{1}^{2}\right) }{\Phi _{1}}}\nonumber \\{} & {} \left( \frac{-\mu +\sqrt{4\rho \sigma -\mu ^{2}}\left( \tan \left( {\sqrt{4\rho \sigma -\mu ^{2}}\varrho }\right) \pm \sec \left( {\sqrt{4\rho \sigma -\mu ^{2}}\varrho }\right) \right) }{2\rho }\right) {,} \end{aligned}$$

68 $$\begin{aligned} \Theta _{1,2,16}(s, t)= & {} \frac{3\zeta ^{2}c_{3}-3\xi ^{2}c_{3}\Lambda _{1}^{2}-2\eta _{1}\Phi _{2}}{6\eta _{1}\Phi _{1}}\mp \sqrt{\frac{2\left( \zeta ^{2}c_{4}-\xi ^{2}c_{4}\Lambda _{1}^{2}\right) }{\Phi _{1}}}\nonumber \\{} & {} \left( \frac{-\mu -\sqrt{4\rho \sigma -\mu ^{2}}\left( \cot \left( {\sqrt{4\rho \sigma -\mu ^{2}}\varrho }\right) \pm \csc \left( {\sqrt{4\rho \sigma -\mu ^{2}}\varrho }\right) \right) }{2\rho }\right) {,} \end{aligned}$$

69 $$\begin{aligned} \Theta _{1,2,17}(s, t)= & {} \frac{3\zeta ^{2}c_{3}-3\xi ^{2}c_{3}\Lambda _{1}^{2}-2\eta _{1}\Phi _{2}}{6\eta _{1}\Phi _{1}}\mp \sqrt{\frac{2\left( \zeta ^{2}c_{4}-\xi ^{2}c_{4}\Lambda _{1}^{2}\right) }{\Phi _{1}}}\nonumber \\{} & {} \left( \frac{-2\mu +\sqrt{4\rho \sigma -\mu ^{2}}\left( \tan {\left( \frac{\sqrt{4\rho \sigma -\mu ^{2}}}{4}\varrho \right) }-\cot {\left( \frac{\sqrt{4\rho \sigma -\mu ^{2}}}{4}\varrho \right) }\right) }{4\rho }\right) {,} \end{aligned}$$

70 $$\begin{aligned} \Theta _{1,2,18}(s, t)= & {} \frac{3\zeta ^{2}c_{3}-3\xi ^{2}c_{3}\Lambda _{1}^{2}-2\eta _{1}\Phi _{2}}{6\eta _{1}\Phi _{1}}\mp \sqrt{\frac{2\left( \zeta ^{2}c_{4}-\xi ^{2}c_{4}\Lambda _{1}^{2}\right) }{\Phi _{1}}}\nonumber \\ {}{} & {} \left( \frac{1}{2\rho }\left( -\mu +\frac{\pm \sqrt{\left( E^{2}-F^{2}\right) \left( 4\rho \sigma -\mu ^{2}\right) }-E\left( \sqrt{4\rho \sigma -\mu ^{2}}\right) \cos \left( \sqrt{4\rho \sigma -\mu ^{2}}\varrho \right) }{E\sin \left( \sqrt{4\rho \sigma -\mu ^{2}}\varrho \right) +F}\right) \right) {,} \end{aligned}$$

71 $$\begin{aligned} \Theta _{1,2,19}(s, t)= & {} \frac{3\zeta ^{2}c_{3}-3\xi ^{2}c_{3}\Lambda _{1}^{2}-2\eta _{1}\Phi _{2}}{6\eta _{1}\Phi _{1}}\mp \sqrt{\frac{2\left( \zeta ^{2}c_{4}-\xi ^{2}c_{4}\Lambda _{1}^{2}\right) }{\Phi _{1}}}\nonumber \\ {}{} & {} \left( \frac{1}{2\rho }\left( -\mu -\frac{\pm \sqrt{\left( E^{2}-F^{2}\right) \left( 4\rho \sigma -\mu ^{2}\right) }-E\left( \sqrt{4\rho \sigma -\mu ^{2}}\right) \sin \left( \sqrt{4\rho \sigma -\mu ^{2}}\varrho \right) }{E\cos \left( \sqrt{4\rho \sigma -\mu ^{2}}\varrho \right) +F}\right) \right) {,} \end{aligned}$$

72 $$\begin{aligned} \Theta _{1,2,20}(s, t)= & {} \frac{3\zeta ^{2}c_{3}-3\xi ^{2}c_{3}\Lambda _{1}^{2}-2\eta _{1}\Phi _{2}}{6\eta _{1}\Phi _{1}}\mp \sqrt{\frac{2\left( \zeta ^{2}c_{4}-\xi ^{2}c_{4}\Lambda _{1}^{2}\right) }{\Phi _{1}}}\nonumber \\ {}{} & {} \left( \frac{2\sigma \cos {\left( \frac{\sqrt{4\rho \sigma -\mu ^{2}}}{2}\varrho \right) }}{\sqrt{4\rho \sigma -\mu ^{2}}\sin {\left( \frac{\sqrt{4\rho \sigma -\mu ^{2}}}{2}\varrho \right) }+\mu \cos {\left( \frac{\sqrt{4\rho \sigma -\mu ^{2}}}{2}\varrho \right) }}\right) {,} \end{aligned}$$

73 $$\begin{aligned} \Theta _{1,2,21}(s, t)= & {} \frac{3\zeta ^{2}c_{3}-3\xi ^{2}c_{3}\Lambda _{1}^{2}-2\eta _{1}\Phi _{2}}{6\eta _{1}\Phi _{1}}\mp \sqrt{\frac{2\left( \zeta ^{2}c_{4}-\xi ^{2}c_{4}\Lambda _{1}^{2}\right) }{\Phi _{1}}}\nonumber \\ {}{} & {} \left( \frac{2\sigma \sin {\left( \frac{\sqrt{4\rho \sigma -\mu ^{2}}}{2}\varrho \right) }}{-\mu \sin {\left( \frac{\sqrt{4\rho \sigma -\mu ^{2}}}{2}\varrho \right) }+\sqrt{4\rho \sigma -\mu ^{2}}\cos {\left( \frac{\sqrt{4\rho \sigma -\mu ^{2}}}{2}\varrho \right) }}\right) {,} \end{aligned}$$

74 $$\begin{aligned} \Theta _{1,2,22}(s, t)= & {} \frac{3\zeta ^{2}c_{3}-3\xi ^{2}c_{3}\Lambda _{1}^{2}-2\eta _{1}\Phi _{2}}{6\eta _{1}\Phi _{1}}\mp \sqrt{\frac{2 \left( \zeta ^{2}c_{4}-\xi ^{2}c_{4}\Lambda _{1}^{2}\right) }{\Phi _{1}}}\nonumber \\ {}{} & {} \left. \left( - \frac{2\sigma \cos { \left( \sqrt{4\rho \sigma -\mu ^{2}}\varrho \right) }}{\sqrt{4\rho \sigma -\mu ^{2}}\sin {\left( \sqrt{4\rho \sigma -\mu ^{2}}\varrho \right) }+\mu \cos {\left( \sqrt{4\rho \sigma -\mu ^{2}}\varrho \right) }\pm \sqrt{4\rho \sigma -\mu ^{2}} } \right) \right) {,} \end{aligned}$$

75 $$\begin{aligned} \Theta _{1,2,23}(s, t)= & {} \frac{3\zeta ^{2}c_{3}-3\xi ^{2}c_{3}\Lambda _{1}^{2}-2\eta _{1}\Phi _{2}}{6\eta _{1}\Phi _{1}}\mp \sqrt{\frac{2\left( \zeta ^{2}c_{4}-\xi ^{2}c_{4}\Lambda _{1}^{2}\right) }{\Phi _{1}}}\nonumber \\{} & {} \left( \frac{2\sigma \sin {\left( \sqrt{4\rho \sigma -\mu ^{2}}\varrho \right) }}{\left( \sqrt{4\rho \sigma -\mu ^{2}}\cos {\left( \sqrt{4\rho \sigma -\mu ^{2}}\varrho \right) }\pm \left( \sqrt{4\rho \sigma -\mu ^{2}}\right) \right) -\mu \sin {\left( \sqrt{4\rho \sigma -\mu ^{2}}\varrho \right) }}\right) {,} \end{aligned}$$

76 $$\begin{aligned} \Theta _{1,2,24}(s, t)= & {} \frac{3\zeta ^{2}c_{3}-3\xi ^{2}c_{3}\Lambda _{1}^{2}-2\eta _{1}\Phi _{2}}{6\eta _{1}\Phi _{1}}\mp \sqrt{\frac{2\left( \zeta ^{2}c_{4}-\xi ^{2}c_{4}\Lambda _{1}^{2}\right) }{\Phi _{1}}}\nonumber \\{} & {} \left( \chi _{24}^{I}(\varrho )=\frac{4\sigma \cos {\left( \frac{\sqrt{4\rho \sigma -\mu ^{2}}\varrho }{4}\right) }\sin {\left( \frac{\sqrt{4\rho \sigma -\mu ^{2}}\varrho }{4}\right) }}{-2\mu \cos {\left( \frac{\sqrt{4\rho \sigma -\mu ^{2}}\varrho }{4}\right) }\sin {\left( \frac{\sqrt{4\rho \sigma -\mu ^{2}}\varrho }{4}\right) }+2\sqrt{\mu ^{2}-4\rho \sigma }\cos ^{2}{\left( \frac{\sqrt{4\rho \sigma -\mu ^{2}}\varrho }{4}\right) }-\left( \sqrt{4\rho \sigma -\mu ^{2}}\right) }\right) {.} \end{aligned}$$

### Case 2

#### Type 1: $\rho \sigma <0$ and $\rho \sigma \ne 0$

77 $$\begin{aligned} \Theta _{2,1,25}(s, t )= & {} \frac{3\zeta ^{2}c_{3}-3\xi ^{2}c_{3}\Lambda _{1}^{2}-2\eta _{1}\Phi _{2}}{6\eta _{1}\Phi _{1}}\mp \sqrt{\frac{2\left( \zeta ^{2}c_{4}-\xi ^{2}c_{4}\Lambda _{1}^{2}\right) }{\Phi _{1}}}\nonumber \\ {}{} & {} \left( -\frac{\pm \sqrt{-2\rho \sigma }+\sqrt{-6\rho \sigma } \tanh {\left( \frac{\sqrt{-6\rho \sigma }\varrho }{2}\right) }}{2\rho }\right) {,} \end{aligned}$$

78 $$\begin{aligned} \Theta _{2,1,26}\left( s, t\right)= & {} \frac{3\zeta ^{2}c_{3}-3\xi ^{2}c_{3}\Lambda _{1}^{2}-2\eta _{1}\Phi _{2}}{6\eta _{1}\Phi _{1}}\mp \sqrt{\frac{2\left( \zeta ^{2}c_{4}-\xi ^{2}c_{4}\Lambda _{1}^{2}\right) }{\Phi _{1}}}\nonumber \\ {}{} & {} \left( -\frac{\pm \sqrt{-2\rho \sigma }+\sqrt{-6\rho \sigma } \coth {\left( \frac{\sqrt{-6\rho \sigma }\varrho }{2}\right) }}{2\rho }\right) {,} \end{aligned}$$

79 $$\begin{aligned} \Theta _{2,1,27}(s, t)= & {} \frac{3\zeta ^{2}c_{3}-3\xi ^{2}c_{3}\Lambda _{1}^{2}-2\eta _{1}\Phi _{2}}{6\eta _{1}\Phi _{1}}\mp \sqrt{\frac{2 \left( \zeta ^{2}c_{4}-\xi ^{2}c_{4}\Lambda _{1}^{2} \right) }{\Phi _{1}}}\nonumber \\ {}{} & {} \left( -\frac{\pm \sqrt{-2\rho \sigma }+\sqrt{-6\rho \sigma } \left( \tanh \left( {\sqrt{-6\rho \sigma }\varrho } \right) \pm \iota \text {sech} \left( {\sqrt{-6\rho \sigma }\varrho } \right) \right) }{2\rho } \right) {,} \end{aligned}$$

80 $$\begin{aligned} \Theta _{2,1,28}(s, t)= & {} \frac{3\zeta ^{2}c_{3}-3\xi ^{2}c_{3}\Lambda _{1}^{2}-2\eta _{1}\Phi _{2}}{6\eta _{1}\Phi _{1}}\mp \sqrt{\frac{2 \left( \zeta ^{2}c_{4}-\xi ^{2}c_{4}\Lambda _{1}^{2} \right) }{\Phi _{1}}}\nonumber \\ {}{} & {} \left( -\frac{\pm \sqrt{-2\rho \sigma }+\sqrt{-6\rho \sigma }\left( \coth \left( {\sqrt{-6\rho \sigma }\varrho } \right) \pm \iota \text {csch} \left( {\sqrt{-6\rho \sigma }\varrho } \right) \right) }{2\rho } \right) {,} \end{aligned}$$

81 $$\begin{aligned} \Theta _{2,1,29}(s, t)= & {} \frac{3\zeta ^{2}c_{3}-3\xi ^{2}c_{3}\Lambda _{1}^{2}-2\eta _{1}\Phi _{2}}{6\eta _{1}\Phi _{1}}\mp \sqrt{\frac{2\left( \zeta ^{2}c_{4}-\xi ^{2}c_{4}\Lambda _{1}^{2}\right) }{\Phi _{1}}}\nonumber \\ {}{} & {} \left( -\frac{\pm \sqrt{-2\rho \sigma }+\sqrt{-6\rho \sigma }\left( \tanh \left( \frac{\sqrt{-6\rho \sigma }\varrho }{4}\right) +\coth \left( \frac{\sqrt{-6\rho \sigma }\varrho }{4}\right) \right) }{4\rho }\right) {,} \end{aligned}$$

82 $$\begin{aligned} \Theta _{2,1,30}(s, t)= & {} \frac{3\zeta ^{2}c_{3}-3\xi ^{2}c_{3}\Lambda _{1}^{2}-2\eta _{1}\Phi _{2}}{6\eta _{1}\Phi _{1}}\mp \sqrt{\frac{2 \left( \zeta ^{2}c_{4}-\xi ^{2}c_{4}\Lambda _{1}^{2}\right) }{\Phi _{1}}}\nonumber \\ {}{} & {} \left( \frac{1}{2\rho }\left( \mp \sqrt{-2\rho \sigma }+\frac{\sqrt{\left( E^{2}+F^{2}\right) \left( -6\rho \sigma \right) }-E\left( \sqrt{-6\rho \sigma }\right) \cosh \left( \sqrt{-6\rho \sigma }\varrho \right) }{E\sinh \left( \sqrt{-6\rho \sigma }\varrho \right) +F}\right) \right) {,} \end{aligned}$$

83 $$\begin{aligned} \Theta _{2,1,31}(s, t)= & {} \frac{3\zeta ^{2}c_{3}-3\xi ^{2}c_{3}\Lambda _{1}^{2}-2\eta _{1}\Phi _{2}}{6\eta _{1}\Phi _{1}}\mp \sqrt{\frac{2 \left( \zeta ^{2}c_{4} -\xi ^{2}c_{4}\Lambda _{1}^{2} \right) }{\Phi _{1}}}\nonumber \\{} & {} \left( \frac{1}{2\rho } \left( \mp \sqrt{-2\rho \sigma }-\frac{\sqrt{\left( F^{2}-E^{2} \right) \left( -6\rho \sigma \right) }+E \left( \sqrt{-6\rho \sigma } \right) \sinh \left( \sqrt{-6\rho \sigma } \varrho \right) }{E\cosh \left( \sqrt{-6\rho \sigma }\varrho \right) +F} \right) \right) {,}\end{aligned}$$

where E and F are non-zero constants that fulfill $F^{2}-E^{2}>0$.

84 $$\begin{aligned} \Theta _{2,1,32}(s, t)= & {} \frac{3\zeta ^{2}c_{3}-3\xi ^{2}c_{3}\Lambda _{1}^{2}-2\eta _{1}\Phi _{2}}{6\eta _{1}\Phi _{1}}\mp \sqrt{\frac{2\left( \zeta ^{2}c_{4}-\xi ^{2}c_{4}\Lambda _{1}^{2}\right) }{\Phi _{1}}}\nonumber \\ {}{} & {} \left( \frac{2\sigma \cosh {\left( \frac{\sqrt{-6\rho \sigma }\varrho }{2}\right) }}{\sqrt{-6\rho \sigma }\sinh {\left( \frac{\sqrt{-6\rho \sigma }\varrho }{2}\right) }\mp \sqrt{-2\rho \sigma }\cosh {\left( \frac{\sqrt{-6\rho \sigma }\varrho }{2}\right) }}\right) {,} \end{aligned}$$

85 $$\begin{aligned} \Theta _{2,1,33}(s, t)= & {} \frac{3\zeta ^{2}c_{3}-3\xi ^{2}c_{3}\Lambda _{1}^{2}-2\eta _{1}\Phi _{2}}{6\eta _{1}\Phi _{1}}\mp \sqrt{\frac{2\left( \zeta ^{2}c_{4}-\xi ^{2}c_{4}\Lambda _{1}^{2}\right) }{\Phi _{1}}}\nonumber \\ {}{} & {} \left( \frac{-2\sigma \sinh {\left( \frac{\sqrt{-6\rho \sigma }\varrho }{2}\right) }}{\pm \sqrt{-2\rho \sigma }\sinh {\left( \frac{\sqrt{-6\rho \sigma }\varrho }{2}\right) }-\sqrt{-6\rho \sigma }\cosh {\left( \frac{\sqrt{-6\rho \sigma }\varrho }{2}\right) }}\right) {,} \end{aligned}$$

86 $$\begin{aligned} \Theta _{2,1,34}(s, t)= & {} \frac{3\zeta ^{2}c_{3}-3\xi ^{2}c_{3}\Lambda _{1}^{2}-2\eta _{1}\Phi _{2}}{6\eta _{1}\Phi _{1}}\mp \sqrt{\frac{2 \left( \zeta ^{2}c_{4}-\xi ^{2}c_{4}\Lambda _{1}^{2} \right) }{\Phi _{1}}}\nonumber \\ {}{} & {} \left( \frac{2\sigma \cosh { \left( \sqrt{-6\rho \sigma }\varrho \right) }}{\sqrt{-6\rho \sigma }\sinh { \left( \sqrt{-6\rho \sigma }\varrho \right) }\mp \sqrt{-2\rho \sigma }\cosh { \left( \sqrt{-6\rho \sigma }\varrho \right) }\pm \iota \left( \sqrt{-6\rho \sigma } \right) } \right) {,} \end{aligned}$$

87 $$\begin{aligned} \Theta _{2,1,35}(s, t)= & {} \frac{3\zeta ^{2}c_{3}-3\xi ^{2}c_{3}\Lambda _{1}^{2}-2\eta _{1}\Phi _{2}}{6\eta _{1}\Phi _{1}}\mp \sqrt{\frac{2 \left( \zeta ^{2}c_{4}-\xi ^{2}c_{4}\Lambda _{1}^{2} \right) }{\Phi _{1}}}\nonumber \\ {}{} & {} \Bigg (\frac{2\sigma \sinh {(\sqrt{-6\rho \sigma }\varrho )}}{\mp \sqrt{-2\rho \sigma }\sinh {(\sqrt{-6\rho \sigma }\varrho )}+\sqrt{-6\rho \sigma }\cosh {(\sqrt{-6\rho \sigma }\varrho )}\pm \iota \sqrt{-6\rho \sigma })} \Bigg ){,} \end{aligned}$$

88 $$\begin{aligned} \Theta _{2,1,36}(s, t)= & {} \frac{3\zeta ^{2}c_{3}-3\xi ^{2}c_{3}\Lambda _{1}^{2}-2\eta _{1}\Phi _{2}}{6\eta _{1}\Phi _{1}}\mp \sqrt{\frac{2\left( \zeta ^{2}c_{4}-\xi ^{2}c_{4}\Lambda _{1}^{2}\right) }{\Phi _{1}}}\nonumber \\ {}{} & {} \left( \frac{4\sigma \cosh {\left( \frac{\sqrt{-6\rho \sigma }\varrho }{4}\right) }\sinh {\left( \frac{\sqrt{-6\rho \sigma }\varrho }{4}\right) }}{\pm 2\sqrt{-2\rho \sigma }\cosh {\left( \frac{\sqrt{-6\rho \sigma }\varrho }{4}\right) }\sinh {\left( \frac{\sqrt{-6\rho \sigma }\varrho }{4}\right) }+2\sqrt{-6\rho \sigma }\cosh ^{2}{\left( \frac{\sqrt{-6\rho \sigma }\varrho }{4}\right) }-\left( \sqrt{-6\rho \sigma }\right) }\right) {.} \end{aligned}$$

### Case 3

#### Type 1: When $c_{2}=1, c_{3}=-\frac{2\alpha _{3}}{\alpha _{1}}, c_{4}=\frac{\alpha _{3}^{2}-\alpha _{2}^{2}}{\alpha _{1}^{2}}$

89 $$\begin{aligned} \Theta _{3,1,37}(s, t) = \frac{3\zeta ^{2}c_{3}-3\xi ^{2}c_{3}\Lambda _{1}^{2}-2\eta _{1}\Phi _{2}}{6\eta _{1}\Phi _{1}}\mp \sqrt{\frac{2 \left( \zeta ^{2}c_{4}-\xi ^{2}c_{4}\Lambda _{1}^{2} \right) }{\Phi _{1}}} \left( \frac{\alpha _{1}sech(\varrho )}{\alpha _{2}+\alpha _{3}sech(\varrho )} \right) {.} \end{aligned}$$

#### Type 2: When $c_{2}=1, c_{3}=-\frac{2\alpha _{3}}{\alpha _{1}}, c_{4}=\frac{\alpha _{3}^{2}+\alpha _{2}^{2}}{\alpha _{1}^{2}}$

90 $$\begin{aligned} \Theta _{3,2,38}(s, t) = \frac{3\zeta ^{2}c_{3}-3\xi ^{2}c_{3}\Lambda _{1}^{2}-2\eta _{1}\Phi _{2}}{6\eta _{1}\Phi _{1}}\mp \sqrt{\frac{2 \left( \zeta ^{2}c_{4}-\xi ^{2}c_{4}\Lambda _{1}^{2} \right) }{\Phi _{1}}} \left( \frac{\alpha _{1}csch(\varrho )}{\alpha _{2}+\alpha _{3}csch(\varrho )} \right) {.} \end{aligned}$$

#### Type 3: When $c_{2}=4, c_{3}=-\frac{4(2\alpha _{2}+\alpha _{4})}{\alpha _{1}}, c_{4}=\frac{\alpha _{3}^{2}+4\alpha _{2}^{2}+4\alpha _{2}\alpha _{4}}{\alpha _{1}^{2}}$

91 $$\begin{aligned} \Theta _{3,3,39}(s, t) = \frac{3\zeta ^{2}c_{3}-3\xi ^{2}c_{3}\Lambda _{1}^{2}-2\eta _{1}\Phi _{2}}{6\eta _{1}\Phi _{1}}\mp \sqrt{\frac{2(\zeta ^{2}c_{4}-\xi ^{2}c_{4}\Lambda _{1}^{2})}{\Phi _{1}}}(\frac{\alpha _{1}sech^{2}(\varrho )}{\alpha _{2}sech^{2}(\varrho )+\alpha _{3}\tanh (\varrho )+\alpha _{4}}){.} \end{aligned}$$

#### Type 4: When $c_{2}=4, c_{3}=-\frac{4(\alpha _{4}-2\alpha _{2})}{\alpha _{1}}, c_{4}=\frac{\alpha _{3}^{2}+4\alpha _{2}^{2}-4\alpha _{2}\alpha _{4}}{\alpha _{1}^{2}}$

92 $$\begin{aligned} \Theta _{3,4,40}(s, t) = \frac{3\zeta ^{2}c_{3}-3\xi ^{2}c_{3}\Lambda _{1}^{2}-2\eta _{1}\Phi _{2}}{6\eta _{1}\Phi _{1}}\mp \sqrt{\frac{2 \left( \zeta ^{2}c_{4}-\xi ^{2}c_{4}\Lambda _{1}^{2} \right) }{\Phi _{1}}} \left( \frac{\alpha _{1}csch^{2}(\varrho )}{\alpha _{2}\coth ^{2}(\varrho )+\alpha _{3}\tanh (\varrho )+\alpha _{4}} \right) {.} \end{aligned}$$

#### Type 5: When $c_{2}=\alpha _{1}^{2}, c_{3}=2\alpha _{1}\alpha _{2}, c_{4}=\alpha _{2}^{2}$

93 $$\begin{aligned} \Theta _{3,5,41}(s, t)= & {} \frac{3\zeta ^{2}c_{3}-3\xi ^{2}c_{3}\Lambda _{1}^{2}-2\eta _{1}\Phi _{2}}{6\eta _{1}\Phi _{1}}\mp \sqrt{\frac{2 \left( \zeta ^{2}c_{4}-\xi ^{2}c_{4}\Lambda _{1}^{2} \right) }{\Phi _{1}}} \left( -\frac{\alpha _{1}\alpha _{3}}{\alpha _{2} \left( \cosh \left( \alpha _{1}\varrho \right) -\sinh \left( \alpha _{1}\varrho \right) +\alpha _{3} \right) } \right) {,} \end{aligned}$$

94 $$\begin{aligned} \Theta _{3,5,42}(s, t)= & {} \frac{3\zeta ^{2}c_{3}-3\xi ^{2}c_{3}\Lambda _{1}^{2}-2\eta _{1}\Phi _{2}}{6\eta _{1}\Phi _{1}}\mp \sqrt{\frac{2(\zeta ^{2}c_{4}-\xi ^{2}c_{4}\Lambda _{1}^{2})}{\Phi _{1}}} \left( -\frac{\alpha _{1}(\sinh (\alpha _{1}\varrho )+\cosh (\alpha _{1}\varrho ))}{\alpha _{2}(\sinh (\alpha _{1}\varrho )+\cosh (\alpha _{1}\varrho )+\alpha _{3})} \right) {.} \end{aligned}$$

#### Type 6: When $c_{2}=-1, c_{3}=\frac{2\alpha _{3}}{\alpha _{1}}, c_{4}=-\frac{\alpha _{3}^{2}-\alpha _{2}^{2}}{\alpha _{1}^{2}}$

95 $$\begin{aligned} \Theta _{3,6,43}(s, t)= & {} \frac{3\zeta ^{2}c_{3}-3\xi ^{2}c_{3}\Lambda _{1}^{2}-2\eta _{1}\Phi _{2}}{6\eta _{1}\Phi _{1}}\mp \sqrt{\frac{2(\zeta ^{2}c_{4}-\xi ^{2}c_{4}\Lambda _{1}^{2})}{\Phi _{1}}} \left( \frac{\alpha _{1}\sec (\varrho )}{\alpha _{2}+\alpha _{3}\sec (\varrho )} \right) {,} \end{aligned}$$

96 $$\begin{aligned} \Theta _{3,6,44}(s, t)= & {} \frac{3\zeta ^{2}c_{3}-3\xi ^{2}c_{3}\Lambda _{1}^{2}-2\eta _{1}\Phi _{2}}{6\eta _{1}\Phi _{1}}\mp \sqrt{\frac{2(\zeta ^{2}c_{4}-\xi ^{2}c_{4}\Lambda _{1}^{2})}{\Phi _{1}}} \left( \frac{\alpha _{1}\csc (\varrho )}{\alpha _{2}+\alpha _{3}\csc (\varrho )} \right) {.} \end{aligned}$$

#### Type 7: When $c_{2}=-4, c_{3}=\frac{4(2\alpha _{2}+\alpha _{4})}{\alpha _{1}}, c_{4}=-\frac{-\alpha _{3}^{2}+4\alpha _{2}^{2}+4\alpha _{2}\alpha _{4}}{\alpha _{1}^{2}}$

97 $$\begin{aligned} \Theta _{3,7,45}(s, t)= & {} \frac{3\zeta ^{2}c_{3}-3\xi ^{2}c_{3}\Lambda _{1}^{2}-2\eta _{1}\Phi _{2}}{6\eta _{1}\Phi _{1}}\mp \sqrt{\frac{2 \left( \zeta ^{2}c_{4}-\xi ^{2}c_{4}\Lambda _{1}^{2} \right) }{\Phi _{1}}}\nonumber \\{} & {} \left( \frac{\alpha _{1}\sec ^{2}(\varrho )}{\alpha _{2}\sec ^{2}(\varrho )+\alpha _{3}\tan (\varrho )+\alpha _{4}}\frac{\alpha _{1}\sec ^{2}(\varrho )}{\alpha _{2}\sec ^{2}(\varrho )+\alpha _{3}\tan (\varrho )+\alpha _{4}} \right) {,} \end{aligned}$$

98 $$\begin{aligned} \Theta _{3,7,46}(s, t)= & {} \frac{3\zeta ^{2}c_{3}-3\xi ^{2}c_{3}\Lambda _{1}^{2}-2\eta _{1}\Phi _{2}}{6\eta _{1}\Phi _{1}}\mp \sqrt{\frac{2(\zeta ^{2}c_{4}-\xi ^{2}c_{4}\Lambda _{1}^{2})}{\Phi _{1}}} \left( \frac{\alpha _{1}\csc ^{2}(\varrho )}{\alpha _{2}\csc ^{2}(\varrho )+\alpha _{3}\cot (\varrho )+\alpha _{4}} \right) {,} \end{aligned}$$

where the constants $\alpha _{1}, \alpha _{2}, \alpha _{3}$ and $\alpha _{4}$ are arbitrary.

### Case 4

#### Type 1: When $c_{0}=1, c_{2}=-(1-q^{2}), c_{4}=q^{2}$

99 $$\begin{aligned} \Theta _{4,1,47}(s, t)= & {} \frac{3\zeta ^{2}c_{3}-3\xi ^{2}c_{3}\Lambda _{1}^{2}-2\eta _{1}\Phi _{2}}{6\eta _{1}\Phi _{1}}\mp \sqrt{\frac{2 \left( \zeta ^{2}c_{4}-\xi ^{2}c_{4}\Lambda _{1}^{2} \right) }{\Phi _{1}}}(cn\varrho ){,} \end{aligned}$$

100 $$\begin{aligned} \Theta _{4,1,48}(s, t)= & {} \frac{3\zeta ^{2}c_{3}-3\xi ^{2}c_{3}\Lambda _{1}^{2}-2\eta _{1}\Phi _{2}}{6\eta _{1}\Phi _{1}}\mp \sqrt{\frac{2(\zeta ^{2}c_{4}-\xi ^{2}c_{4}\Lambda _{1}^{2})}{\Phi _{1}}} \left( \frac{cn\varrho }{cd\varrho } \right) {.} \end{aligned}$$

#### Type 2: When $c_{0}=1-q^{2}, c_{2}=2q^{2}-1, c_{4}=-q^{2}$

101 $$\begin{aligned} \Theta _{4,2,49}(s, t) = \frac{3\zeta ^{2}c_{3}-3\xi ^{2}c_{3}\Lambda _{1}^{2}-2\eta _{1}\Phi _{2}}{6\eta _{1}\Phi _{1}}\mp \sqrt{\frac{2(\zeta ^{2}c_{4}-\xi ^{2}c_{4}\Lambda _{1}^{2})}{\Phi _{1}}}(cn\varrho ){.} \end{aligned}$$

#### Type 3: When $c_{0}=q^{2}-1, c_{2}=2-q^{2}, c_{4}=-1$

102 $$\begin{aligned} \Theta _{4,3,50}(s, t) = \frac{3\zeta ^{2}c_{3}-3\xi ^{2}c_{3}\Lambda _{1}^{2}-2\eta _{1}\Phi _{2}}{6\eta _{1}\Phi _{1}}\mp \sqrt{\frac{2(\zeta ^{2}c_{4}-\xi ^{2}c_{4}\Lambda _{1}^{2})}{\Phi _{1}}}(dn\varrho ){.} \end{aligned}$$

#### Type 4: When $c_{0}=q^{2}, c_{2}=-(1+q^{2}), c_{4}=1$

103 $$\begin{aligned} \Theta _{4,4,51}(s, t) = \frac{3\zeta ^{2}c_{3}-3\xi ^{2}c_{3}\Lambda _{1}^{2}-2\eta _{1}\Phi _{2}}{6\eta _{1}\Phi _{1}}\mp \sqrt{\frac{2(\zeta ^{2}c_{4}-\xi ^{2}c_{4}\Lambda _{1}^{2})}{\Phi _{1}}}(rn\varrho )^{-1}{,} \end{aligned}$$

104 $$\begin{aligned} \Theta _{4,4,52}(s, t) = \frac{3\zeta ^{2}c_{3}-3\xi ^{2}c_{3}\Lambda _{1}^{2}-2\eta _{1}\Phi _{2}}{6\eta _{1}\Phi _{1}}\mp \sqrt{\frac{2(\zeta ^{2}c_{4}-\xi ^{2}c_{4}\Lambda _{1}^{2})}{\Phi _{1}}} \left( \frac{dn\varrho }{cn\varrho } \right) {.} \end{aligned}$$

#### Type 5: When $c_{0}=-q^{2}, c_{2}=2q^{2}-1, c_{4}=1-q^{2}$

105 $$\begin{aligned} \Theta _{4,5,53}(s, t) = \frac{3\zeta ^{2}c_{3}-3\xi ^{2}c_{3}\Lambda _{1}^{2}-2\eta _{1}\Phi _{2}}{6\eta _{1}\Phi _{1}}\mp \sqrt{\frac{2(\zeta ^{2}c_{4}-\xi ^{2}c_{4}\Lambda _{1}^{2})}{\Phi _{1}}}(cn\varrho )^{-1}{.} \end{aligned}$$

#### Type 6: When $c_{0}=-1, c_{2}=2-q^{2}, c_{4}=q^{2}-1$

106 $$\begin{aligned} \Theta _{4,6,54}(s, t) = \frac{3\zeta ^{2}c_{3}-3\xi ^{2}c_{3}\Lambda _{1}^{2}-2\eta _{1}\Phi _{2}}{6\eta _{1}\Phi _{1}}\mp \sqrt{\frac{2(\zeta ^{2}c_{4}-\xi ^{2}c_{4}\Lambda _{1}^{2})}{\Phi _{1}}}(dn\varrho )^{-1}{.} \end{aligned}$$

#### Type 7: When $c_{0}=1, c_{2}=2-q^{2}, c_{4}=1-q^{2}$

107 $$\begin{aligned} \Theta _{4,7,55}(s, t) = \frac{3\zeta ^{2}c_{3}-3\xi ^{2}c_{3}\Lambda _{1}^{2}-2\eta _{1}\Phi _{2}}{6\eta _{1}\Phi _{1}}\mp \sqrt{\frac{2(\zeta ^{2}c_{4}-\xi ^{2}c_{4}\Lambda _{1}^{2})}{\Phi _{1}}} \left( \frac{rn\varrho }{cn\varrho } \right) {.} \end{aligned}$$

#### Type 8: When $c_{0}=1, c_{2}=2q^{2}-1, c_{4}=-q^{2}(1-q^{2})$

108 $$\begin{aligned} \Theta _{4,8,56}(s, t) = \frac{3\zeta ^{2}c_{3}-3\xi ^{2}c_{3}\Lambda _{1}^{2}-2\eta _{1}\Phi _{2}}{6\eta _{1}\Phi _{1}}\mp \sqrt{\frac{2(\zeta ^{2}c_{4}-\xi ^{2}c_{4}\Lambda _{1}^{2})}{\Phi _{1}}} \left( \frac{rn\varrho }{dn\varrho } \right) {.} \end{aligned}$$

#### Type 9: When $c_{0}=1-q^{2}, c_{2}=2-q^{2}, c_{4}=1$

109 $$\begin{aligned} \Theta _{4,9,57}(s, t) = \frac{3\zeta ^{2}c_{3}-3\xi ^{2}c_{3}\Lambda _{1}^{2}-2\eta _{1}\Phi _{2}}{6\eta _{1}\Phi _{1}}\mp \sqrt{\frac{2(\zeta ^{2}c_{4}-\xi ^{2}c_{4}\Lambda _{1}^{2})}{\Phi _{1}}} \left( \frac{cn\varrho }{rn\varrho } \right) {.} \end{aligned}$$

#### Type 10: When $c_{0}=-q^{2}(1-q^{2}), c_{2}=2q^{2}-1, c_{4}=1$

110 $$\begin{aligned} \Theta _{4,10,58}(s, t) = \frac{3\zeta ^{2}c_{3}-3\xi ^{2}c_{3}\Lambda _{1}^{2}-2\eta _{1}\Phi _{2}}{6\eta _{1}\Phi _{1}}\mp \sqrt{\frac{2(\zeta ^{2}c_{4}-\xi ^{2}c_{4}\Lambda _{1}^{2})}{\Phi _{1}}} \left( \frac{dn\varrho }{rn\varrho } \right) {.} \end{aligned}$$

#### Type 11: When $c_{0}=\frac{1}{4}, c_{2}=\frac{1-2q^{2}}{2}, c_{4}=frac{1}{4}$

111 $$\begin{aligned} \Theta _{4,11,59}(s, t) = \frac{3\zeta ^{2}c_{3}-3\xi ^{2}c_{3}\Lambda _{1}^{2}-2\eta _{1}\Phi _{2}}{6\eta _{1}\Phi _{1}}\mp \sqrt{\frac{2(\zeta ^{2}c_{4}-\xi ^{2}c_{4}\Lambda _{1}^{2})}{\Phi _{1}}}(nr\varrho \pm cr\varrho ){.} \end{aligned}$$

#### Type 12: When $c_{0}=\frac{1-q^{2}}{4}, c_{2}=\frac{1+q^{2}}{2}, c_{4}=\frac{1-q^{2}}{4}$

112 $$\begin{aligned} \Theta _{4,12,60}(s, t) = \frac{3\zeta ^{2}c_{3}-3\xi ^{2}c_{3}\Lambda _{1}^{2}-2\eta _{1}\Phi _{2}}{6\eta _{1}\Phi _{1}}\mp \sqrt{\frac{2(\zeta ^{2}c_{4}-\xi ^{2}c_{4}\Lambda _{1}^{2})}{\Phi _{1}}}(nc\varrho \pm rc\varrho ){.} \end{aligned}$$

#### Type 13: When $c_{0}=\frac{q^{2}}{4}, c_{2}=\frac{q^{2}-2}{2}, c_{4}=\frac{1}{4}$

113 $$\begin{aligned} \Theta _{4,13,61}(s, t) = \frac{3\zeta ^{2}c_{3}-3\xi ^{2}c_{3}\Lambda _{1}^{2}-2\eta _{1}\Phi _{2}}{6\eta _{1}\Phi _{1}}\mp \sqrt{\frac{2(\zeta ^{2}c_{4}-\xi ^{2}c_{4}\Lambda _{1}^{2})}{\Phi _{1}}}(nr\varrho \pm dr\varrho ){.} \end{aligned}$$

#### Type 14: When $c_{0}=\frac{q^{2}}{4}, c_{2}=\frac{q^{2}-2}{2}, c_{4}=\frac{q^{2}}{4}$

114 $$\begin{aligned} \Theta _{4,14,62}(s, t) = \frac{3\zeta ^{2}c_{3}-3\xi ^{2}c_{3}\Lambda _{1}^{2}-2\eta _{1}\Phi _{2}}{6\eta _{1}\Phi _{1}}\mp \sqrt{\frac{2(\zeta ^{2}c_{4}-\xi ^{2}c_{4}\Lambda _{1}^{2})}{\Phi _{1}}}(rn\varrho \pm \iota cr\varrho ){,} \end{aligned}$$

where *q* is the JEF satisfying $0 \le q \le 1$. Equations (99)–(114) can be stated as follows when $q \rightarrow 1$, JEFs degenerate into hyperbolic functions, which is illustrated in Table 2.

115 $$\begin{aligned} \Theta _{4,1,63}(s, t)= & {} \frac{3\zeta ^{2}c_{3}-3\xi ^{2}c_{3}\Lambda _{1}^{2}-2\eta _{1}\Phi _{2}}{6\eta _{1}\Phi _{1}}\mp \sqrt{\frac{2(\zeta ^{2}c_{4}-\xi ^{2}c_{4}\Lambda _{1}^{2})}{\Phi _{1}}}(sech\varrho ){,} \end{aligned}$$

116 $$\begin{aligned} \Theta _{4,1,64}(s, t)= & {} \frac{3\zeta ^{2}c_{3}-3\xi ^{2}c_{3}\Lambda _{1}^{2}-2\eta _{1}\Phi _{2}}{6\eta _{1}\Phi _{1}}\mp \sqrt{\frac{2(\zeta ^{2}c_{4}-\xi ^{2}c_{4}\Lambda _{1}^{2})}{\Phi _{1}}}(sech\varrho ){,} \end{aligned}$$

117 $$\begin{aligned} \Theta _{4,2,65}(s, t)= & {} \frac{3\zeta ^{2}c_{3}-3\xi ^{2}c_{3}\Lambda _{1}^{2}-2\eta _{1}\Phi _{2}}{6\eta _{1}\Phi _{1}}\mp \sqrt{\frac{2(\zeta ^{2}c_{4}-\xi ^{2}c_{4}\Lambda _{1}^{2})}{\Phi _{1}}}(sech\varrho ){,} \end{aligned}$$

118 $$\begin{aligned} \Theta _{4,3,66}(s, t)= & {} \frac{3\zeta ^{2}c_{3}-3\xi ^{2}c_{3}\Lambda _{1}^{2}-2\eta _{1}\Phi _{2}}{6\eta _{1}\Phi _{1}}\mp \sqrt{\frac{2(\zeta ^{2}c_{4}-\xi ^{2}c_{4}\Lambda _{1}^{2})}{\Phi _{1}}}(sech\varrho ){,} \end{aligned}$$

119 $$\begin{aligned} \Theta _{4,4,67}(s, t)= & {} \frac{3\zeta ^{2}c_{3}-3\xi ^{2}c_{3}\Lambda _{1}^{2}-2\eta _{1}\Phi _{2}}{6\eta _{1}\Phi _{1}}\mp \sqrt{\frac{2(\zeta ^{2}c_{4}-\xi ^{2}c_{4}\Lambda _{1}^{2})}{\Phi _{1}}}(\tanh \varrho )^{-1}{,} \end{aligned}$$

120 $$\begin{aligned} \Theta _{4,4,68}(s, t)= & {} \frac{3\zeta ^{2}c_{3}-3\xi ^{2}c_{3}\Lambda _{1}^{2}-2\eta _{1}\Phi _{2}}{6\eta _{1}\Phi _{1}}\mp \sqrt{\frac{2(\zeta ^{2}c_{4}-\xi ^{2}c_{4}\Lambda _{1}^{2})}{\Phi _{1}}} \left( \frac{sech\varrho }{sech\varrho } \right) {,} \end{aligned}$$

121 $$\begin{aligned} \Theta _{4,5,69}(s, t)= & {} \frac{3\zeta ^{2}c_{3}-3\xi ^{2}c_{3}\Lambda _{1}^{2}-2\eta _{1}\Phi _{2}}{6\eta _{1}\Phi _{1}}\mp \sqrt{\frac{2(\zeta ^{2}c_{4}-\xi ^{2}c_{4}\Lambda _{1}^{2})}{\Phi _{1}}}(sech\varrho )^{-1}{,} \end{aligned}$$

122 $$\begin{aligned} \Theta _{4,6,70}(s, t)= & {} \frac{3\zeta ^{2}c_{3}-3\xi ^{2}c_{3}\Lambda _{1}^{2}-2\eta _{1}\Phi _{2}}{6\eta _{1}\Phi _{1}}\mp \sqrt{\frac{2(\zeta ^{2}c_{4}-\xi ^{2}c_{4}\Lambda _{1}^{2})}{\Phi _{1}}}(sech\varrho )^{-1}{,} \end{aligned}$$

123 $$\begin{aligned} \Theta _{4,7,71}(s, t)= & {} \frac{3\zeta ^{2}c_{3}-3\xi ^{2}c_{3}\Lambda _{1}^{2}-2\eta _{1}\Phi _{2}}{6\eta _{1}\Phi _{1}}\mp \sqrt{\frac{2(\zeta ^{2}c_{4}-\xi ^{2}c_{4}\Lambda _{1}^{2})}{\Phi _{1}}} \left( \frac{\tanh \varrho }{sech\varrho } \right) {,} \end{aligned}$$

124 $$\begin{aligned} \Theta _{4,8,72}(s, t)= & {} \frac{3\zeta ^{2}c_{3}-3\xi ^{2}c_{3}\Lambda _{1}^{2}-2\eta _{1}\Phi _{2}}{6\eta _{1}\Phi _{1}}\mp \sqrt{\frac{2(\zeta ^{2}c_{4}-\xi ^{2}c_{4}\Lambda _{1}^{2})}{\Phi _{1}}} \left( \frac{\sinh \varrho }{sech\varrho } \right) {,} \end{aligned}$$

125 $$\begin{aligned} \Theta _{4,9,73}(s, t)= & {} \frac{3\zeta ^{2}c_{3}-3\xi ^{2}c_{3}\Lambda _{1}^{2}-2\eta _{1}\Phi _{2}}{6\eta _{1}\Phi _{1}}\mp \sqrt{\frac{2(\zeta ^{2}c_{4}-\xi ^{2}c_{4}\Lambda _{1}^{2})}{\Phi _{1}}} \left( \frac{sech\varrho }{\tanh \varrho } \right) {,} \end{aligned}$$

126 $$\begin{aligned} \Theta _{4,10,74}(s, t)= & {} \frac{3\zeta ^{2}c_{3}-3\xi ^{2}c_{3}\Lambda _{1}^{2}-2\eta _{1}\Phi _{2}}{6\eta _{1}\Phi _{1}}\mp \sqrt{\frac{2(\zeta ^{2}c_{4}-\xi ^{2}c_{4}\Lambda _{1}^{2})}{\Phi _{1}}} \left( \frac{sech\varrho }{\tanh \varrho } \right) {,} \end{aligned}$$

127 $$\begin{aligned} \Theta _{4,11,75}(s, t)= & {} \frac{3\zeta ^{2}c_{3}-3\xi ^{2}c_{3}\Lambda _{1}^{2}-2\eta _{1}\Phi _{2}}{6\eta _{1}\Phi _{1}}\mp \sqrt{\frac{2(\zeta ^{2}c_{4}-\xi ^{2}c_{4}\Lambda _{1}^{2})}{\Phi _{1}}} \left( \coth \varrho \pm csch\varrho \right) {,} \end{aligned}$$

128 $$\begin{aligned} \Theta _{4,12,76}(s, t)= & {} \frac{3\zeta ^{2}c_{3}-3\xi ^{2}c_{3}\Lambda _{1}^{2}-2\eta _{1}\Phi _{2}}{6\eta _{1}\Phi _{1}}\mp \sqrt{\frac{2(\zeta ^{2}c_{4}-\xi ^{2}c_{4}\Lambda _{1}^{2})}{\Phi _{1}}} \left( \cosh \varrho \pm \sinh \varrho \right) {,} \end{aligned}$$

129 $$\begin{aligned} \Theta _{4,13,77}(s, t)= & {} \frac{3\zeta ^{2}c_{3}-3\xi ^{2}c_{3}\Lambda _{1}^{2}-2\eta _{1}\Phi _{2}}{6\eta _{1}\Phi _{1}}\mp \sqrt{\frac{2(\zeta ^{2}c_{4}-\xi ^{2}c_{4}\Lambda _{1}^{2})}{\Phi _{1}}} \left( \coth \varrho \pm csch\varrho \right) {,} \end{aligned}$$

130 $$\begin{aligned} \Theta _{4,14,78}(s, t)= & {} \frac{3\zeta ^{2}c_{3}-3\xi ^{2}c_{3}\Lambda _{1}^{2}-2\eta _{1}\Phi _{2}}{6\eta _{1}\Phi _{1}}\mp \sqrt{\frac{2(\zeta ^{2}c_{4}-\xi ^{2}c_{4}\Lambda _{1}^{2})}{\Phi _{1}}} \left( \tanh \varrho \pm \iota csch\varrho \right) {,} \end{aligned}$$

likewise, when $q \rightarrow 0$, JEFs degenerate into trigonometric functions, as shown in Table 3, Eqs. (99)–(114) can be expressed as subsequently

131 $$\begin{aligned} \Theta _{4,1,79}(s, t)= & {} \frac{3\zeta ^{2}c_{3}-3\xi ^{2}c_{3}\Lambda _{1}^{2}-2\eta _{1}\Phi _{2}}{6\eta _{1}\Phi _{1}}\mp \sqrt{\frac{2(\zeta ^{2}c_{4}-\xi ^{2}c_{4}\Lambda _{1}^{2})}{\Phi _{1}}}(\cos \varrho ){,} \end{aligned}$$

132 $$\begin{aligned} \Theta _{4,1,80}(s, t)= & {} \frac{3\zeta ^{2}c_{3}-3\xi ^{2}c_{3}\Lambda _{1}^{2}-2\eta _{1}\Phi _{2}}{6\eta _{1}\Phi _{1}}\mp \sqrt{\frac{2(\zeta ^{2}c_{4}-\xi ^{2}c_{4}\Lambda _{1}^{2})}{\Phi _{1}}} \end{aligned}$$

133 $$\begin{aligned} \Theta _{4,2,81}(s, t)= & {} \frac{3\zeta ^{2}c_{3}-3\xi ^{2}c_{3}\Lambda _{1}^{2}-2\eta _{1}\Phi _{2}}{6\eta _{1}\Phi _{1}}\mp \sqrt{\frac{2(\zeta ^{2}c_{4}-\xi ^{2}c_{4}\Lambda _{1}^{2})}{\Phi _{1}}}(\cos \varrho ){,} \end{aligned}$$

134 $$\begin{aligned} \Theta _{4,3,82}(s, t)= & {} \frac{3\zeta ^{2}c_{3}-3\xi ^{2}c_{3}\Lambda _{1}^{2}-2\eta _{1}\Phi _{2}}{6\eta _{1}\Phi _{1}}\mp \sqrt{\frac{2(\zeta ^{2}c_{4}-\xi ^{2}c_{4}\Lambda _{1}^{2})}{\Phi _{1}}}{,} \end{aligned}$$

135 $$\begin{aligned} \Theta _{4,4,83}(s, t)= & {} \frac{3\zeta ^{2}c_{3}-3\xi ^{2}c_{3}\Lambda _{1}^{2}-2\eta _{1}\Phi _{2}}{6\eta _{1}\Phi _{1}}\mp \sqrt{\frac{2(\zeta ^{2}c_{4}-\xi ^{2}c_{4}\Lambda _{1}^{2})}{\Phi _{1}}}(\sin \varrho )^{-1}{,} \end{aligned}$$

136 $$\begin{aligned} \Theta _{4,4,84}(s, t)= & {} \frac{3\zeta ^{2}c_{3}-3\xi ^{2}c_{3}\Lambda _{1}^{2}-2\eta _{1}\Phi _{2}}{6\eta _{1}\Phi _{1}}\mp \sqrt{\frac{2(\zeta ^{2}c_{4}-\xi ^{2}c_{4}\Lambda _{1}^{2})}{\Phi _{1}}} \left( \frac{dn\varrho }{\cos \varrho } \right) {,} \end{aligned}$$

137 $$\begin{aligned} \Theta _{4,5,85}(s, t)= & {} \frac{3\zeta ^{2}c_{3}-3\xi ^{2}c_{3}\Lambda _{1}^{2}-2\eta _{1}\Phi _{2}}{6\eta _{1}\Phi _{1}}\mp \sqrt{\frac{2(\zeta ^{2}c_{4}-\xi ^{2}c_{4}\Lambda _{1}^{2})}{\Phi _{1}}}(\cos \varrho )^{-1}{,} \end{aligned}$$

138 $$\begin{aligned} \Theta _{4,6,86}(s, t)= & {} \frac{3\zeta ^{2}c_{3}-3\xi ^{2}c_{3}\Lambda _{1}^{2}-2\eta _{1}\Phi _{2}}{6\eta _{1}\Phi _{1}}\mp \sqrt{\frac{2(\zeta ^{2}c_{4}-\xi ^{2}c_{4}\Lambda _{1}^{2})}{\Phi _{1}}}{,} \end{aligned}$$

139 $$\begin{aligned} \Theta _{4,7,87}(s, t)= & {} \frac{3\zeta ^{2}c_{3}-3\xi ^{2}c_{3}\Lambda _{1}^{2}-2\eta _{1}\Phi _{2}}{6\eta _{1}\Phi _{1}}\mp \sqrt{\frac{2(\zeta ^{2}c_{4}-\xi ^{2}c_{4}\Lambda _{1}^{2})}{\Phi _{1}}} \left( \frac{\sin \varrho }{\cos \varrho } \right) {,} \end{aligned}$$

140 $$\begin{aligned} \Theta _{4,8,88}(s, t)= & {} \frac{3\zeta ^{2}c_{3}-3\xi ^{2}c_{3}\Lambda _{1}^{2}-2\eta _{1}\Phi _{2}}{6\eta _{1}\Phi _{1}}\mp \sqrt{\frac{2 \left( \zeta ^{2}c_{4}-\xi ^{2}c_{4}\Lambda _{1}^{2} \right) }{\Phi _{1}}}(\sin \varrho ){,} \end{aligned}$$

141 $$\begin{aligned} \Theta _{4,9,89}(s, t)= & {} \frac{3\zeta ^{2}c_{3}-3\xi ^{2}c_{3}\Lambda _{1}^{2}-2\eta _{1}\Phi _{2}}{6\eta _{1}\Phi _{1}}\mp \sqrt{\frac{2(\zeta ^{2}c_{4}-\xi ^{2}c_{4}\Lambda _{1}^{2})}{\Phi _{1}}} \left( \frac{\cos \varrho }{\sin \varrho } \right) {,} \end{aligned}$$

142 $$\begin{aligned} \Theta _{4,10,90}(s, t)= & {} \frac{3\zeta ^{2}c_{3}-3\xi ^{2}c_{3}\Lambda _{1}^{2}-2\eta _{1}\Phi _{2}}{6\eta _{1}\Phi _{1}}\mp \sqrt{\frac{2(\zeta ^{2}c_{4}-\xi ^{2}c_{4}\Lambda _{1}^{2})}{\Phi _{1}}} \left( \frac{1}{\sin \varrho } \right) {,} \end{aligned}$$

143 $$\begin{aligned} \Theta _{4,11,91}(s, t)= & {} \frac{3\zeta ^{2}c_{3}-3\xi ^{2}c_{3}\Lambda _{1}^{2}-2\eta _{1}\Phi _{2}}{6\eta _{1}\Phi _{1}}\mp \sqrt{\frac{2(\zeta ^{2}c_{4}-\xi ^{2}c_{4}\Lambda _{1}^{2})}{\Phi _{1}}} \left( \csc \varrho \pm \cot \varrho \right) {,} \end{aligned}$$

144 $$\begin{aligned} \Theta _{4,12,92}(s, t)= & {} \frac{3\zeta ^{2}c_{3}-3\xi ^{2}c_{3}\Lambda _{1}^{2}-2\eta _{1}\Phi _{2}}{6\eta _{1}\Phi _{1}}\mp \sqrt{\frac{2(\zeta ^{2}c_{4}-\xi ^{2}c_{4}\Lambda _{1}^{2})}{\Phi _{1}}} \left( \sec \varrho \pm \tan \varrho \right) {,} \end{aligned}$$

145 $$\begin{aligned} \Theta _{4,13,93}(s, t)= & {} \frac{3\zeta ^{2}c_{3}-3\xi ^{2}c_{3}\Lambda _{1}^{2}-2\eta _{1}\Phi _{2}}{6\eta _{1}\Phi _{1}}\mp \sqrt{\frac{2(\zeta ^{2}c_{4}-\xi ^{2}c_{4}\Lambda _{1}^{2})}{\Phi _{1}}} \left( \csc \varrho \pm \csc \varrho \right) {,} \end{aligned}$$

146 $$\begin{aligned} \Theta _{4,14,94}(s, t)= & {} \frac{3\zeta ^{2}c_{3}-3\xi ^{2}c_{3}\Lambda _{1}^{2}-2\eta _{1}\Phi _{2}}{6\eta _{1}\Phi _{1}}\mp \sqrt{\frac{2(\zeta ^{2}c_{4}-\xi ^{2}c_{4}\Lambda _{1}^{2})}{\Phi _{1}}} \left( \sin \varrho \pm \iota \cot \varrho \right) {,} \end{aligned}$$

where $$\varrho = \xi s + \zeta t$$.

## Graphical findings and discussion

The visual representations of the D-CDNA model is examined in this section. When trying to construct the exact TW solutions, the visual behavior related to the D-CDNA model is developed using the MEFSEM for various parametric assumptions. To evaluate the structural design of D-CDNA model with manipulating the parameters set, the Mathematica 13.2 computer software is utilized. By adjusting the settings of the parameter, the graphs appearance of D-CDNA can be modified. Along with adding to the 3D plots and related 2D line graphs, we additionally provided contour plots for straightforward understanding. Distinct wave patterns can be developed via assigning distinct values for the parameters. By implementing MEFSEM into practice, numerous solutions, such as complexiton, kink wave, dark or anti-bell, V, anti-Z and singular wave shapes soliton solutions can be encountered.

**Figure 2:**
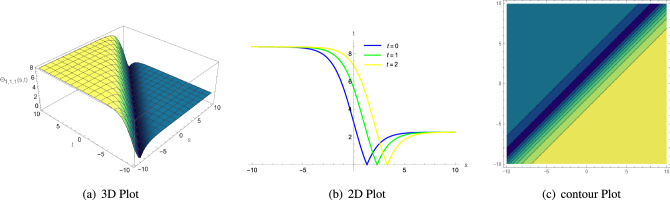
(**a**) A 3D plot of $\Theta _{1,1,1}(s, t)$ given in Eq. (53) is kink wave soliton, (**b**) analogous 2D line graphs for numerous values of *t* and (**c**) associated contour plot when $\zeta = 4$, $\rho =0.01$, $\Lambda _{1}=1$, $\xi =1$, $\Phi _{2}=1$, $\Phi _{1}=1$, $\sigma =1$ and $\mu =1$.

**Figure 3:**
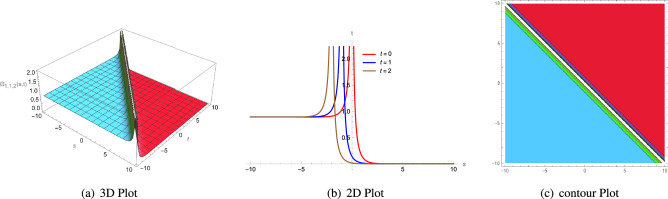
(**a**) A 3D plot of $\Theta _{1,1,2}(s, t)$ given in Eq. (54) is singular wave soliton, (**b**) analogous 2D line graphs for numerous values of *t* and (**c**) associated contour plot when $\zeta = 0.5$, $\rho =0.1$, $\Lambda _{1}=1$, $\xi =0.01$, $\Phi _{2}=10$, $\Phi _{1}=10$, $\sigma =0$ and $\mu =1.5$.

**Figure 4:**
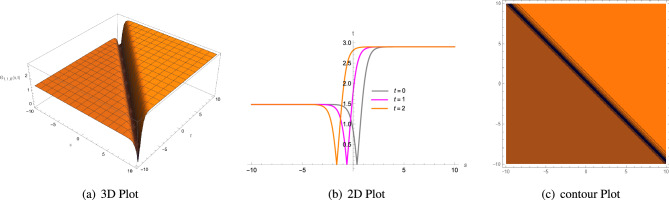
(**a**) A 3D plot of $\Theta _{1,1,8}(s, t)$ given in Eq. (60) is complexiton shape soliton, (**b**) analogous 2D line graphs for numerous values of *t* and (**c**) associated contour plot when $\zeta = 1.5$, $\rho =2$, $\Lambda _{1}=1$, $\xi =1$, $\Phi _{2}=1$, $\Phi _{1}=5$, $\sigma =1$ and $\mu =3.5$.

**Figure 5:**
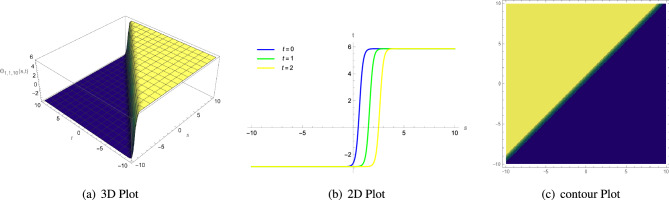
(**a**) A 3D plot of $\Theta _{1,1,10}(s, t)$ given in Eq. (62) is anti-Z shape soliton, (**b**) analogous 2D line graphs for numerous values of *t* and (**c**) associated contour plot when $\zeta = 3$, $\rho =2$, $\Lambda _{1}=1$, $\xi =1$, $\Phi _{2}=1$, $\Phi _{1}=5$, $\sigma =1$ and $\mu =3$.

**Figure 6:**
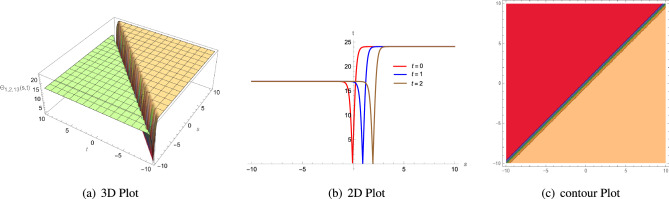
(**a**) A 3D plot of $\Theta _{1,2,13}(s, t)$ given in Eq. (65) is anti-bell shape or dark soliton soliton, (**b**) analogous 2D line graphs for numerous values of *t* and (**c**) associated contour plot when $\zeta = 4$, $\rho =10$, $\Lambda _{1}=2$, $\xi =1$, $\Phi _{2}=1$, $\Phi _{1}=1$, $\sigma =1$ and $\mu =1$.

**Figure 7:**
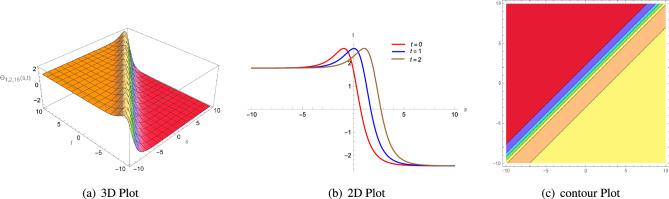
(**a**) A 3D plot of $\Theta _{1,2,15}(s, t)$ given in Eq. (67) is kink wave soliton, (**b**) analogous 2D line graphs for numerous values of *t* and (**c**) associated contour plot when $\zeta = 3.5$, $\rho =0.01$, $\Lambda _{1}=5$, $\xi =1$, $\Phi _{2}=1$, $\Phi _{1}=1$, $\sigma =1$ and $\mu =3.5$.

**Figure 8:**
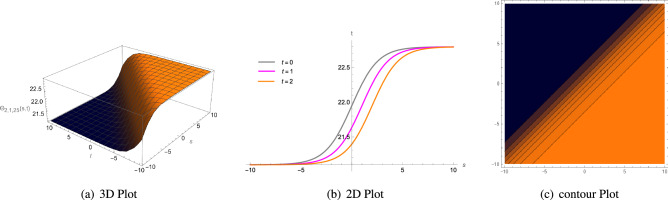
(**a**) A 3D plot of $\Theta _{2,1,25}(s, t)$ given in Eq. (77) is kink wave soliton, (**b**) analogous 2D line graphs for numerous values of *t* and (**c**) associated contour plot when $\zeta = 5$, $\rho =0.1$, $\Lambda _{1}=1$, $\xi =2$, $\Phi _{2}=1$, $\Phi _{1}=10$, $\sigma =1$ and $\mu =10$.

**Figure 9:**
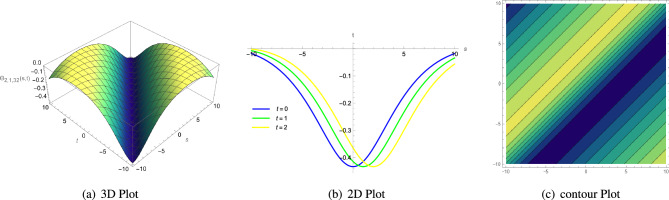
(**a**) A 3D plot of $\Theta _{2,1,32}(s, t)$ given in Eq. (84) is V-shape soliton, (**b**) analogous 2D line graphs for numerous values of *t* and (**c**) associated contour plot when $\zeta = 5$, $\rho =0.01$, $\Lambda _{1}=3.5$, $\xi =2.5$, $\Phi _{2}=1$, $\Phi _{1}=5$, $\sigma =1$ and $\mu =3$.

Figure 2 exhibits the 3D, 2D at multiple values of *t* and contour plots of the solution $\Theta _{1,1,1}(s, t)$ given in Eq. (53) while considering parametric values $\zeta = 4$, $\rho =0.01$, $\Lambda _{1}=1$, $\xi =1$, $\Phi _{2}=1$, $\Phi _{1}=1$, $\sigma =1$ and $\mu =1$, which displays a kink wave soliton. Figure 3 exhibits the 3D, 2D at multiple values of *t* and contour plots of the solution $\Theta _{1,1,2}(s, t)$ given in Eq. (54) while considering parametric values $\zeta = 0.5$, $\rho =0.1$, $\Lambda _{1}=1$, $\xi =0.01$, $\Phi _{2}=10$, $\Phi _{1}=10$, $\sigma =0$ and $\mu =1.5$, which displays a singular shape soliton. Figure 4 exhibits the 3D, 2D at multiple values of *t* and contour plots of the solution $\Theta _{1,1,8}(s, t)$ given in Eq. (60) while considering parametric values $\zeta = 1.5$, $\rho =2$, $\Lambda _{1}=1$, $\xi =1$, $\Phi _{2}=1$, $\Phi _{1}=5$, $\sigma =1$ and $\mu =3.5$, which displays a complexitons shape soliton. Figure 5 exhibits the 3D, 2D at multiple values of *t* and contour plots of the solution $\Theta _{1,1,10}(s, t)$ given in Eq. (62) while considering parametric values $\zeta = 3$, $\rho =2$, $\Lambda _{1}=1$, $\xi =1$, $\Phi _{2}=1$, $\Phi _{1}=5$, $\sigma =1$ and $\mu =3$, which displays a anti-Z shape soliton. Figure 6 exhibits the 3D, 2D at multiple values of *t* and contour plots of the solution $\Theta _{1,2,13}(s, t)$ given in Eq. (65) while considering parametric values $\zeta = 4$, $\rho =10$, $\Lambda _{1}=2$, $\xi =1$, $\Phi _{2}=1$, $\Phi _{1}=1$, $\sigma =1$ and $\mu =1$, which displays a anti-bell shape or dark soliton soliton. Figure 7 exhibits the 3D, 2D at multiple values of *t* and contour plots of the solution $\Theta _{1,2,15}(s, t)$ given in Eq. (67) while considering parametric values $\zeta = 3.5$, $\rho =0.01$, $\Lambda _{1}=5$, $\xi =1$, $\Phi _{2}=1$, $\Phi _{1}=1$, $\sigma =1$ and $\mu =3.5$, which displays a kink wave soliton. Figure 8 exhibits the 3D, 2D at multiple values of *t* and contour plots of the solution $\Theta _{2,1,25}(s, t)$ given in Eq. (77) while considering parametric values $\zeta = 5$, $\rho =0.1$, $\Lambda _{1}=1$, $\xi =2$, $\Phi _{2}=1$, $\Phi _{1}=10$, $\sigma =1$ and $\mu =10$, which displays a kink wave soliton. Figure 9 exhibits the 3D, 2D at multiple values of *t* and contour plots of the solution $\Theta _{2,1,32}(s, t)$ given in Eq. (84) while considering parametric values $\zeta = 5$, $\rho =0.01$, $\Lambda _{1}=3.5$, $\xi =2.5$, $\Phi _{2}=1$, $\Phi _{1}=5$, $\sigma =1$ and $\mu =3$, which displays a V-shape soliton.

## Conclusion

In the present research, by utilizing the MEFSEM to find exact TW solutions to D-CDNA model, that, due to its biological basis, constitutes one of the many intriguing issues in contemporary biophysics. This technique frequently produces deeper, original, general solutions and accurate findings than rival methods, which is its main advantage. Several soliton solutions have been developed for a range of parametric factors. Using MEFSEM, a vast array of novel solutions have been created, such as the complexiton, kink wave, dark or anti-bell, V, anti-Z and singular wave shapes soliton solutions. Numerous examples of soliton solutions can be seen visually. In pure and applied mathematics, soliton solution are widely used especially in disciplines like differential equations, algebraic and differential geometry, Lie groups, and Lie algebras. Soliton-admitting models possess extensive mathematical architecture and characteristics. An infinite number of conservation laws and related symmetries especially are closely linked to the integrability of the these models, became one of their fundamental characteristics. An further characteristic is the presence of a Hamiltonian or bi-Hamiltonian form, which enables the analysis and description of a system without adequately the need for explicit solution of the related models. In contrast to bright or bell shape solitons, dark or anti-bell shape solitons in fiber lasers exhibit greater stability and resistance to distortion in noise-filled environments. Therefore, dark or anti-bell shape solitons are frequently employed in the disciplines of nonlinear optics, optic exchanges, and optic sensor. The kink soliton is a semi-local NL pattern that shows a steep curve in the spectrum across an unchanged bottom height and numerous fading oscillation tails, comparable to other soliton forms. Intensive short-pulse characteristics are influenced by the waveform structure because kink solitons cause self-steepness or NL impacts in NL fibers. Kink solitons have real-world applications as optical logic devices or as polarized switches among two distinct domains. Mathematica 13.2, a straightforward mathematical software, is employed to validate the reliability of the aforementioned soliton solutions findings. Additionally, there are 2D and 3D visualizations that show how the noticed soliton solutions behave dynamically. Because the contour plots enable in comprehending the dynamical characteristics and trends presented by these soliton solutions, they also feature in this paper. The findings rendered it obvious that some of the soliton solutions listed are distinct and had never been seen before. Furthermore, the MEFSEM could potentially be utilized for NLPDEs in diverse areas, hence boosting its practicality as an instrument for further research endeavors. The results of the present research will provide ideas and impetus for future discussions in the NL sciences, particularly those with biological applications. The method’s ease of use render it a significant addition for the study of NLPDEs and could unlock the door to further advances in this discipline. Since, there exists no single method for resolving the analytical solution of the NLPDE in the domain of integrable systems. A general symbolic computing approach for the analytical solution of a nonlinear partial differential equation is made possible by the implied neural network-based symbol calculation method, which also establishes the groundwork for a universal symbolic calculation method for analytical expressions. This might deliver some novel insights into how the model examined in this publication could be resolved in further research employing this method.

## Data Availability

All data generated or analyzed during this study are included in this manuscript.
